# Polar protein Wag31 both activates and inhibits cell wall metabolism at the poles and septum

**DOI:** 10.3389/fmicb.2022.1085918

**Published:** 2023-01-12

**Authors:** Neda Habibi Arejan, Delfina Ensinck, Lautaro Diacovich, Parthvi Bharatkumar Patel, Samantha Y. Quintanilla, Arash Emami Saleh, Hugo Gramajo, Cara C. Boutte

**Affiliations:** ^1^Department of Biology, University of Texas at Arlington, Arlington, TX, United States; ^2^Laboratory of Physiology and Genetics of Actinomycetes, Instituto de Biología Molecular y Celular de Rosario (IBR-CONICET), Facultad de Ciencias Bioquímicas y Farmacéuticas, Universidad Nacional de Rosario, Rosario, Argentina; ^3^Department of Civil Engineering, University of Texas at Arlington, Arlington, TX, United States

**Keywords:** *Mycobacterium*, polar growth regulation, petidoglycan synthesis, DivIVA, intracellular membrane domain (IMD), MurG, ACCase complex, FtsI

## Abstract

Mycobacterial cell elongation occurs at the cell poles; however, it is not clear how cell wall insertion is restricted to the pole or how it is organized. Wag31 is a pole-localized cytoplasmic protein that is essential for polar growth, but its molecular function has not been described. In this study we used alanine scanning mutagenesis to identify Wag31 residues involved in cell morphogenesis. Our data show that Wag31 helps to control proper septation as well as new and old pole elongation. We have identified key amino acid residues involved in these essential functions. Enzyme assays revealed that Wag31 interacts with lipid metabolism by modulating acyl-CoA carboxylase (ACCase) activity. We show that Wag31 does not control polar growth by regulating the localization of cell wall precursor enzymes to the Intracellular Membrane Domain, and we also demonstrate that phosphorylation of Wag31 does not substantively regulate peptidoglycan metabolism. This work establishes new regulatory functions of Wag31 in the mycobacterial cell cycle and clarifies the need for new molecular models of Wag31 function.

## Introduction

In order to grow, bacteria need to expand the existing cell wall without disturbing its integrity. The peptidoglycan of the cell wall is critical for maintenance of cell shape in most bacteria, and is made of sugar chains crosslinked by small peptides that are assembled just outside of the cell membrane. Many rod shaped organisms expand the peptidoglycan network all along the lateral walls through the activity of membrane-anchored peptidoglycan synthases and their regulators (Zhao et al., 2017; Egan et al., 2020). Mycobacteria, as well as many other Actinomycetes and some Alphaproteobacteria, restrict peptidoglycan expansion to the cell poles (Thanky et al., 2007; Brown et al., 2012). In mycobacteria, the old pole elongates continuously, but there is a delay in initiation of elongation at the new pole – the one most recently created by cell division (Hannebelle et al., 2020) – which results in asymmetric cells (Joyce et al., 2012; Rego et al., 2017; Hannebelle et al., 2020).

The molecular mechanisms that control polar cell envelope expansion are not well described. In this paper we dissect some of the functions of Wag31 (AKA DivIVA, Rv2145c, MSMEG_4217), which is an essential protein for polar growth in mycobacteria (Kang et al., 2008; Melzer et al., 2018). Wag31 is a mycobacterial ortholog of DivIVA. DivIVA proteins are found in Firmicutes and Actinobacteria (Supplementary Figure S1) and they typically localize to the poles and/or septum. They function to recruit and regulate other factors involved in cell division, chromosome segregation and cell wall synthesis (Marston and Errington, 1999; Valbuena et al., 2007; van Baarle et al., 2013; Eswaramoorthy et al., 2014; Sieger and Bramkamp, 2015).

Wag31 is localized at the cell poles (Nguyen et al., 2007; Meniche et al., 2014) and at the septum just before division (Santi et al., 2013). In addition to its role in polar growth, it also helps with chromosome segregation by recruiting the segregation factor ParA to the poles (Pióro et al., 2018). Wag31 is not required for cell wall metabolism, but it is needed to direct this metabolism to the poles (Melzer et al., 2022), though it is not known how it performs this function.

*Mycobacterium smegmatis* (*Msmeg*) does not regulate polar cell wall synthesis by restricting its essential peptidoglycan transglycosylases to the poles, as some other pole-growing species do (Valbuena et al., 2007; Cameron et al., 2014; Sieger and Bramkamp, 2015; Sher et al., 2021), since both RodA and PBP1 are delocalized around the entire cell instead of restricted to the poles (Melzer et al., 2022). However, *Msmeg* does restrict synthesis of many cell wall precursor enzymes near the poles, through their association with the Intracellular Membrane Domain (IMD), a chemically distinct region of the plasma membrane found at the sub polar regions in growing mycobacterial cells (Hayashi et al., 2016; Melzer et al., 2022). Depletion of Wag31 delocalizes the IMD (García-Heredia et al., 2021), though it is not clear whether Wag31 regulates this membrane domain directly, or how the IMD contributes to polar elongation.

Wag31 has been shown to interact with AccA3, and FtsI, but the role of these interactions in regulating cell wall expansion is unknown (Mukherjee et al., 2009; Meniche et al., 2014; Xu et al., 2014). FtsI is a transpeptidase involved in peptidoglycan synthesis at the septum (Mukherjee et al., 2009; Boes et al., 2019), while AccA3 is a common subunit of the essential Acyl-CoA Carboxylase (ACCase) complexes involved in the first steps of lipid synthesis (Gago et al., 2018). The subunit composition of these ACCase complexes determines their preference for using either acetyl-CoA (ACC activity) or propionyl-CoA (PCC activity) as substrates, to yield either malonyl-or methylmalonyl-CoA, respectively, which are used as the elongation units for the synthesis of straight-chain or branched-chain fatty acids-containing lipids. The specificity constant of ACCase 5 (formed by the AccA3, AccD5 and AccE5 subunits) is five time higher for propionyl-CoA compared with acetyl-CoA (Gago et al., 2006) while ACCase 6 (constituted by the AccA3 and AccD6 subunits) has a high preference for acetyl-CoA as a substrate (Daniel et al., 2007).

Here, we obtained and characterized a series of point mutants of *wag31* that exhibit diverse phenotypes, indicating that Wag31 has separate roles in regulating elongation at the new and old poles, and septation. We also show that while phosphorylation of Wag31 does moderately affect polar growth, it is not a critical regulator of this process. Furthermore, our data indicate that Wag31’s role in polar growth is not mediated through controlling the localization of IMD enzymes, or regulating FtsI *via* Wag31 residues NSD46-48. However, critical point mutants of Wag31 allowed us to demonstrate that it does have a role in regulating ACCase complex activity.

## Materials and methods

### Bacterial strains and culture conditions

All *Msmeg* strains were grown in liquid culture in 7H9 (BD, Sparks, MD) medium supplemented with 0.2% glycerol, 0.05% Tween 80, and ADC (5 g/l albumin, 2 g/l dextrose, 0.85 g/l NaCl, 0.003 g/l catalase). For most experiments, *Msmeg* strains were grown on LB Lennox plates. DH5a, TOP10, and XL1-Blue *E.coli* cells were used for cloning. For *E. coli*, antibiotics concentrations were: kanamycin – 50 μg/ml; hygromycin – 100 μg/ml; nourseothricin – 40 μg/ml; Zeocin – 25 μg/ml. For *Msme*g, antibiotic concentrations were: kanamycin – 25 μg/ml; hygromycin – 50 μg/ml; nourseothricin – 20 μg/ml; trimethoprim – 50 μg/ml; Zeocin – 20 μg/ml.

### Growth curves

Strains were grown to logarithmic phase in 7H9 with appropriate antibiotics, then diluted to OD600 = 0.1 without antibiotics in a 96 well plate. A Synergy Neo2 linear Multi-Mode Reader was used to shake the plates continuously for 18 h at 37°C, and read OD every 15 min. To find the doubling time for each strain, the raw data were analyzed with exponential growth equation model using GraphPad Prism (version 9.1.2). Optical density reflects total cell material, not cell number (Schaechter et al., 1958), so it is a better predictor of differences in elongation rate than septation rate.

### Strain construction

To build the allele replacement strains, first *wag31*_Msm_ under its native promotor was cloned into a kanamycin-marked L5 integrating vector and transformed into *Msmeg* mc^2^155. Then, *wag31* at its native locus was replaced with hygromycin resistance cassette by dsDNA recombineering (van Kessel and Hatfull, 2007). This cassette was oriented so the hygR promoter drives expression of downstream genes in the operon. Deletion of *wag31* from the genome was confirmed with PCR checks. *wag31*_Msm_ at the L5 site was swapped with *wag31*_Mtb_ alleles in a nourseothricin-marked L5-integrating vector using L5-phage site allelic exchange as described (Pashley and Parish, 2003). The transformants were screened by antibiotic resistance in order to confirm allele swap vectors containing *wag31*_Mtb_ mutants were created using PCR stitching. All vectors were made using Gibson cloning (Gibson et al., 2009). All strains, plasmids and primers used are listed in supplemental material part (Supplementary Tables S1–S3).

### Mycobacterial protein fragment complementation (M-PFC) assay

*Msmeg* strains carrying Mycobacterial protein fragment complementation (M-PFC) assay vectors were grown to logarithmic phase and struck on 7H11 agar (BD Difco™, Thermo Fisher Scientific) with or without trimethoprim at 50 μg/ml as described (Singh et al., 2006). Plates were incubated at 37°C for 4 days, and then photographed.

### Immunoblot analysis

*Msmeg* strains carrying either M-PFC or swap allele vectors were grown to logarithmic phase (OD600 = 0.5–0.7), pelleted, and lysed by bead beating (Disruptor Beads 0.1 mm from Disruptor Genie®, Mini-Beadbeater-16, Biospect). Supernatants were separated by SDS-PAGE and transferred onto polyvinyl difluoride (PVDF) membranes (GE Healthcare) and blocked with BSA for 1 h. The blot was probed with a-C-terminal-DHFR rabbit antibody (1: 1000 Sigma-Aldrich, D0942), Wag31 antibody (1:10000 Thermo Fisher custom made peptide-induced antibody, epitope (KPPIGKRGYNEDEVDAFLD)), and goat anti-rabbit IgG HRP-conjugated secondary antibody (1:10,000, Thermo Fisher Scientific 31,460) in PBST. α-Rpob was used as loading control (10:10000, Thermo Fisher Scientific, MA1-25425). All bands were quantified using Fiji and normalized to the controls.

### Microscopy

All microscopy was performed on three biological replicate cultures of each strain using a Nikon Ti-2 widefield epifluorescence microscope with a Photometrics Prime 95B camera and a Plan Apo 100x, 1.45-numerical-aperture (NA) lens objective. Cells were taken from logarithmic phase culture in 7H9 and immobilized on 1.5% agarose pads made with Hdb media. To detect GFPmut3 and Venus signal, a filter cube with a 470/40 nm excitation filter, a 525/50 nm emission filter and a 495 nm dichroic mirror was used. To detect mCherry2B signal, a filter cube with a 560/40 nm excitation filter, a 630/70 nm emission filter and a 585 nm dichroic mirror was used. To detect HADA, a filter cube with a 350/50 nm excitation filter, a 460/50 nm emission filter, and a 400 nm dichroic mirror was used. Image analysis was performed using MicrobeJ (Ducret et al., 2016) to make cell ROIs and extract fluorescence data from them. Fluorescence intensity data from MicrobeJ was further analyzed using bespoke MATLAB code (see supplement).

### Cell staining

Cultures were stained with 1 μg/ml HADA (R&D systems) for 15 min with rolling and incubation at 37°C. Stained cultures were then pelleted and washed in 7H9 before imaging. For the elongation assay, cells after 15 min of HADA staining were resuspended into 7H9 for 1.5 h of rolling and incubation at 37°C, then stained with 1 μg/ml NADA (R&D systems) for 2 min at room temperature, washed and resuspended in 7H9 before imaging.

### ACC/PCC assay

ACC and PCC activities in cell-free extracts and in *in vitro* reconstituted complexes were measured by following the incorporation of radioactive HCO_3_ into acid non-volatile material (Hunaiti and Kolattukudy, 1982; Bramwell et al., 1996; Diacovich et al., 2002). The reaction mixture contained 100 mM potassium phosphate, pH 8.0, 300 μg of BSA, 3 mM ATP, 5 mM MgCl_2_, 50 mM NaH^14^CO_3_ (specific activity 200 μCi mmol^−1^ (740 kBq mmol^−1^)), 0.5 mM substrate (acetyl-CoA or propionyl-CoA), and 30 or 100 μg of cell-free protein extract in a total reaction volume of 100 μl. The reaction was initiated by the addition of NaH^14^CO_3_, allowed to proceed at 30°C for 15 min, and stopped with 200 μl of 6 M HCl. The contents of the tubes were then evaporated to dryness at 95°C. The residue was resuspended in 100 μl of water, 1 ml of Optiphase liquid scintillation medium (Wallac Oy) was added, and ^14^C radioactivity determined in a Beckman scintillation liquid counter. Nonspecific CO_2_ fixation by crude extracts was assayed in the absence of substrate. One unit of enzyme activity catalyzed the incorporation of 1 μmol of ^14^C into acid-stable products/min.

### ACCase5 and 6 activity assays from purified components

ACCase5 and 6 were reconstituted from purified components using equimolar amounts of the *α* (AccA3) and *β* (0.8 μM AccD5 or 1.6 μM AccD6) subunits. For the ACCase5 complex reconstitution AccE5 subunit was also included in a final concentration of 1.6 μM. After incubating the complexes for 15 min at room temperature, Wag31 was added in a molar ratio of 2:1 to AccA3. The activity was quantified using the ACC/PCC Assay Radioactive Method. The subunits of ACCases were purified as described (Gago et al., 2006). For Wag31 purification, the plasmid pET28-Wag31 was transformed into the *E. coli* strain BL21 (DE3) star, and the protein synthesis was induced by addition of 0.5 mM IPTG. H_6_-Wag31 was purified using a Ni Sepharose column (GE Healthcare) and aliquots containing Wag 31 were stored in presence of 0.2% Tween-20.

## Results

### Wag31 has distinct roles in several steps of the cell cycle

**Figure 1:**
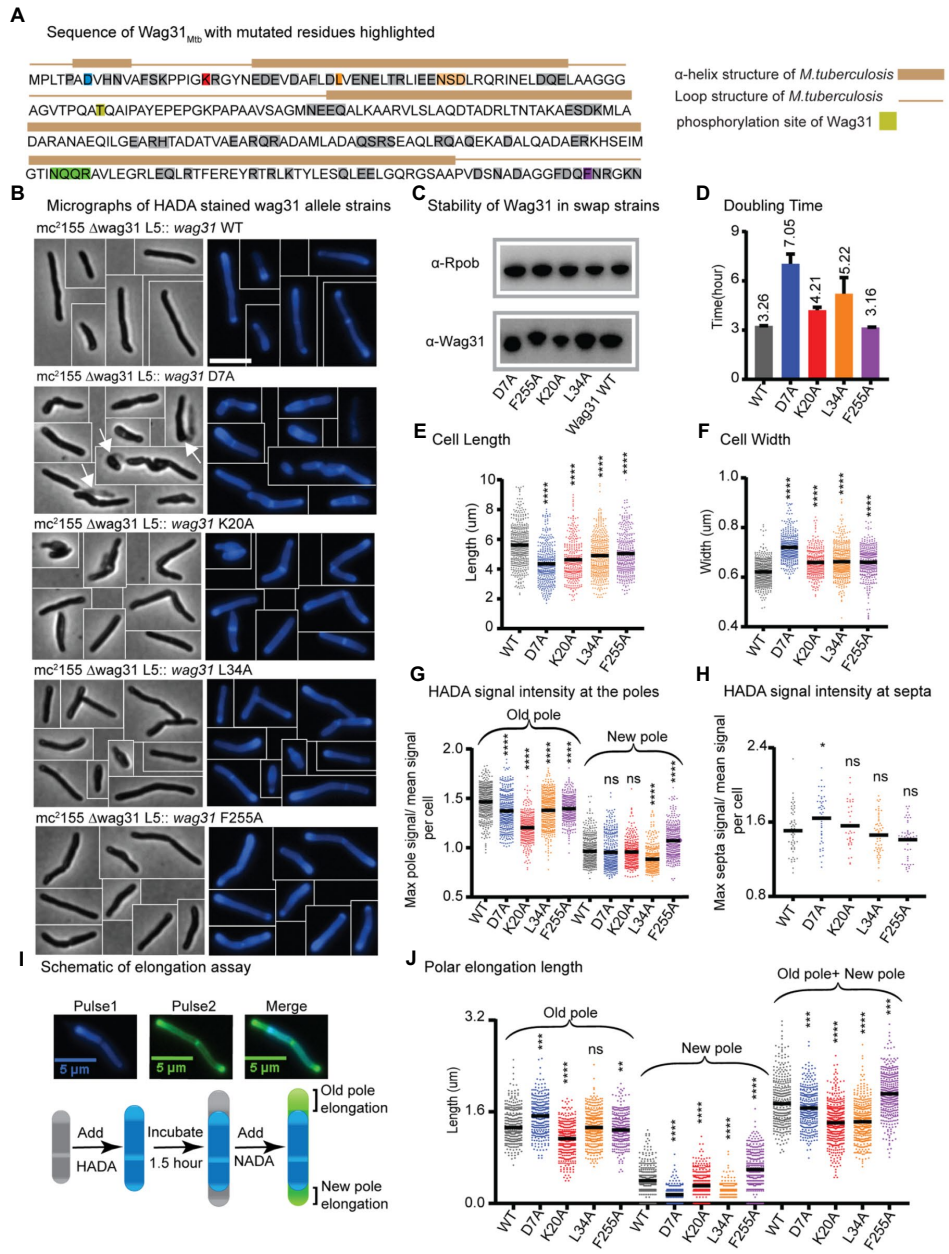
Wag31 has multiple distinct roles in both elongation and division. **(A)** Sequence of Wag31 from *Mycobacterium tuberculosis*. The residues mutated in the alanine scanning experiments are highlighted. **(B)** Phase (left) and fluorescence (right) images of *Msmeg wag31* allele strains stained with HADA. The scale bar is 5 microns, and it applies to all images. Several cells from different images of the same strain are cropped together. White arrows indicate the ghosts in D7A **(C)** Western blots of Wag31 WT and mutant proteins in *wag31* allele strains using α-Wag31 antibody. The epitope used to raise the antibodies is KPPIGKRGYNEDEVDAFLD, of which K is K20. Thus, the decreased signal seen in the K20A strain may be due to decreased association of the antibody against the mutant protein. RpoB serves as a loading control. **(D)** Doubling times of *Msmeg* cells expressing *wag31* WT or alanine mutants. The means (on top of bars) are an average of three biological replicates. Error bars represent SD. **(E)** Cell lengths of the *wag31* allele strains. Black bars are at the mean. **(F)** Cell widths of the *wag31* allele strains. Black bars are at the mean. **(G)** Relative polar and septal **(H)** HADA intensity of *wag31* allele strains. Equivalent to the maximum signal at one pole divided by the mean signal of that cell. **(I)** Schematic of elongation assay dual-FDAA staining method to measure elongation. The brackets indicate the measurements made. **(J)** Length of polar elongation in the *wag31* allele strains, as measured by the elongation assay in I. Black bar is at the median. ns, *p* > 0.05, *p = < 0.05, **p = < 0.005, ***p = <0.0005, ****p = <0.0001. All *p*-values were calculated by one-way ANOVA, Dunnett’s multiple comparisons test, with comparisons of mutants to the wild-type in each case. All microscopy experiments were performed on at least 100 cells from each of three independent replicate strains.

Depletion of Wag31 leads to arrest of polar elongation, loss of pole structure, (Kang et al., 2008; Jani et al., 2010) and delocalization of peptidoglycan and mycomembrane metabolism (Melzer et al., 2018). To dissect Wag31’s functional roles in these processes, we built *Msmeg* strains with *wag31_Mtb_* WT replaced by alanine mutants of *wag31_Mtb_*, using allele swapping with vectors that integrate at the L5 phage integration site (Pashley and Parish, 2003; Figure 1; Supplementary Figures S3–S6). We characterized phenotypes of allele swap mutants through growth curves, microscopy of fluorescent D-amino acid-stained cells, and a test of polar elongation. We tested the stability of the mutated Wag31 proteins by western blotting, and only characterized mutants with stable Wag31 (Figures 1C, 3C; Supplementary Figures S2, S3B). We were unable to swap some of the mutants, thus residues P2, T4, K15, and EQR 210–3 appear to be essential for Wag31 function (Supplementary Figure S4C).

**Figure 2:**
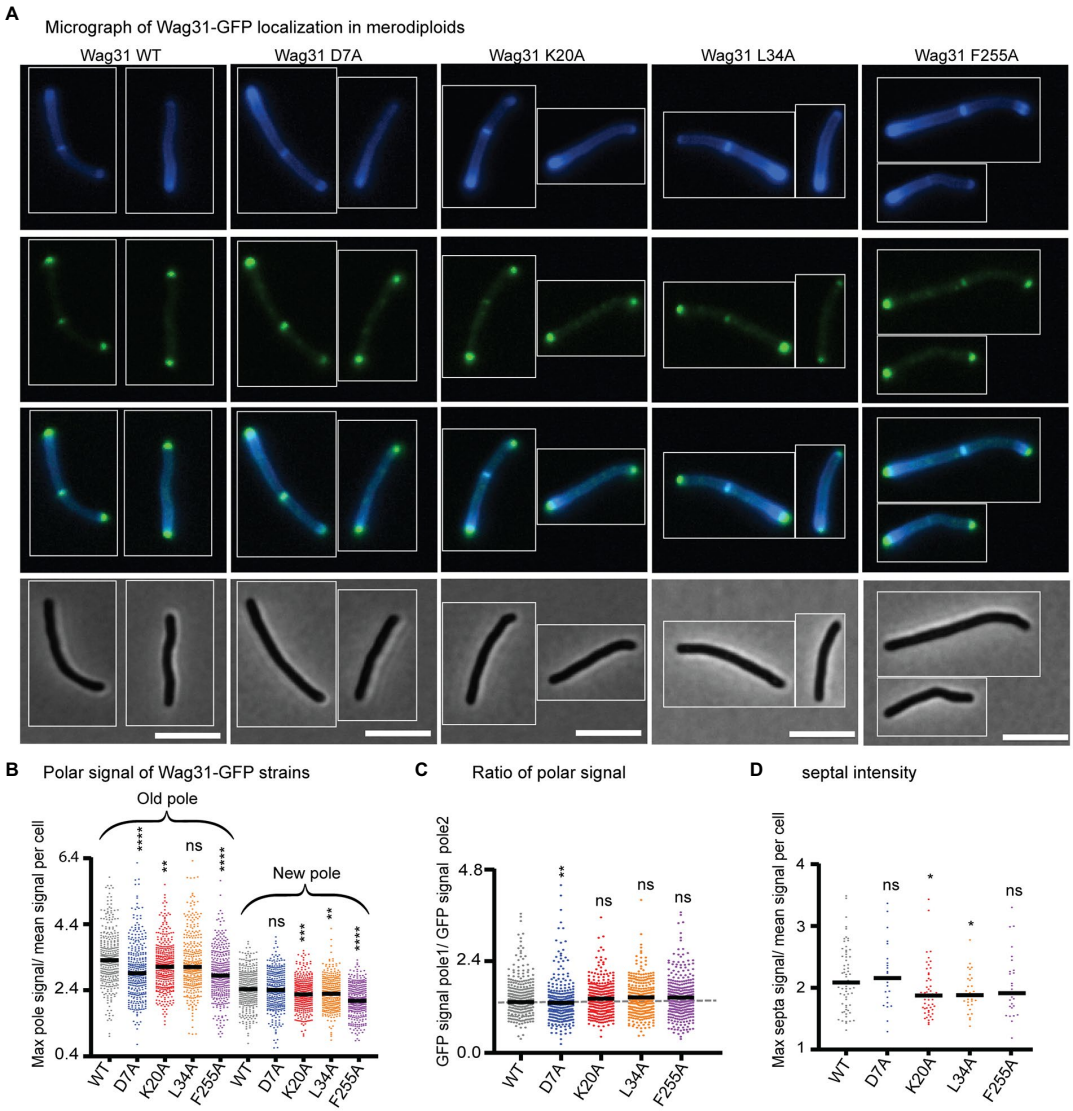
Wag31 mutant proteins localize normally in merodiploid strains. **(A)** Micrographs of WT Msmeg expressing Wag31-GFPmut3 constructs with the indicated mutations. Top: HADA; second: GFP; third: merged; bottom: phase. The scale bar is 5 microns, and it applies to all images. **(B)** Relative polar intensity of GFPmut3 signal from cells in A. **(C)** Ratio of normalized signal at the old pole over normalized signal at the new pole, from B. **(D)** Relative septal intensity of GFPmut3 signal from cells in A. ns, *p* > 0.05, *p = <0.05, **p = <0.005, ***p = <0.0005, ****p = <0.0001. All p-values **(B)**, **(C)** were calculated by one-way ANOVA, Dunnett’s multiple comparisons test and the *p*-values **(D)** were calculated by the Welch’s *t*-test.

Most of the strains with mutations at the N-terminus of Wag31 exhibit slowed growth, while the majority of strains with mutations in the middle and C-terminus of the protein grow as fast as the strain expressing WT Wag31 (Figure 1D; Supplementary Figure S4A). Microscopy experiments show that many of the mutant cells are short and/or wide (Figures 1E,F; Supplementary Figure S4A). Most of the *wag31*_Mtb_ mutant strains have higher signal than the WT after staining with the fluorescent D-alanine HADA (Kuru et al., 2012; Supplementary Figures S5A, S6A, S8, S9), which may result from de-repressed peptidoglycan metabolism or increased cell wall permeability. To compare the localization of peptidoglycan metabolism between strains, we calculated relative HADA intensity at the poles and septa which is the ratio of the maximum intensity at each region to the mean intensity of the cell. Because the older pole almost always stains more brightly by HADA (Baranowski et al., 2018); throughout we assume that the brighter pole is the old pole.

Most mutants had lower relative HADA signal than the WT at the old pole, and none have higher signal (Figure 1G; Supplementary Figure S5B). At the new pole, most mutants stain like the WT, but some are brighter and others dimmer (Figure 1H; Supplementary Figure S5C). These data indicate that mutants of Wag31 can have different impacts on the peptidoglycan metabolism at the two cell poles. Relative septal HADA intensity is increased in a few, while it is decreased in others (Supplementary Figure S6B), suggesting that Wag31 affects peptidoglycan synthesis at the septa as well. We also observe that the septal placement is shifted toward one pole or the other in some mutants (Supplementary Figure S6C).

Previous work showed cell bending when Wag31-eGFP replaces native Wag31 (Meniche et al., 2014). Here, we observed a similar curved cell morphology in the *wag31* NQQR199-202AAAA mutant (Supplementary Figures S3A,C,D). Although the *wag31* NQQR199-202AAAA mutant has slightly decreased relative HADA intensity at the old pole (Supplementary Figure S3F), it elongates like the Wag31 WT at both poles (Supplementary Figure S3H). We surmise that the Wag31-eGFP C-terminal fusion and these mutations may disorder the Wag31 homo-oligomer in a way that could cause cell wall insertion to be uneven around the circumference of the cell, leading to slight polar bulging and curved cells (Supplementary Figure S3).

A variety of different phenotypes indicative of assorted functional roles for Wag31 were apparent across the collection of mutants. We chose for further study four strains with mutations in conserved amino acid residues (Supplementary Figure S1), and which represent a range of phenotypes: D7A, K20A, L34A, and F255A.

### The *wag31* D7A mutant exhibits dysregulated septation

The *wag31* D7A strain grows slowly, and the cells are short and wide (Figures 1B, D–F). There is a slight decrease in relative HADA intensity, compared to WT, at both poles (Figure 1G), but relative HADA intensity is significantly increased at the septa (Figure 1H). We performed an elongation assay with two colors of fluorescent D-amino acids to determine if the dimmer polar HADA staining and short cell length was due to decreased elongation compared to WT. We first stained the cells with blue fluorescent HADA, then outgrew them without stain for 1.5 h, then re-stained them with the green fluorescent D-alanine NADA, and measured the length of the poles that are green and not blue (Figure 1I). Our data (Figure 1J) show that the net elongation defect (7%) does not account for the substantially decreased cell length (22%; Figure 1E), indicating that division must be initiated at a shorter cell length in this strain. The relative septal HADA staining suggests that there is more peptidoglycan metabolism at the septum than in the WT cells (Figure 1H; Supplementary Figure S6B) and that septal staining occurs in shorter cells (Supplementary Figure S8). This cell division may occur earlier, which could lead to the unusual number of ghosts in the *wag31* D7A phase images (Figure 1B). These data suggest that the *wag31* D7A mutation causes septation to occur in an uncontrolled manner. We therefore conclude that Wag31 has a role in controlling septation.

We localized Wag31 D7A-GFPmut3 in a merodiploid strain – because the allele swaps were not viable – and find that it localizes like the Wag31 WT-GFPmut3 at the poles and septum (Figure 2), which allows us to infer that Wag31 D7A can associate normally with WT Wag31 at the polar focus.

### Wag31 regulates polar elongation in different ways

The N-terminus of Wag31 is highly conserved and has been structurally characterized (Oliva et al., 2010; Choukate and Chaudhuri, 2020), while the C-terminus of Wag31 is less well conserved and characterized. In DivIVA from *Bacillus subtilis*, the amino acid F17 is involved in interaction with the membrane at the cell pole (Oliva et al., 2010). In *Msmeg*, K20 is in a comparable position as F17 in DivIVA*_Bsub_* in the structure (Choukate and Chaudhuri, 2020).

We found that mutations in two N-terminal residues, K20 and L34, and a C-terminal residue F255 of Wag31 lead to mis-regulation of polar growth (Figure 1). Point mutations in K20A and L34A cause defects in growth rate, while F255A does not (Figure 1D). All three mutants have short cell lengths (Figures 1B,E).

To determine whether the short cells result from decreased elongation or activated septation, we performed elongation assays (Figures 1I,J). We found that the defect in the *wag31* K20A elongation (22% less than WT) is similar to the defect in cell length (18%), indicating that this mutant is defective in elongation, especially at the old pole (Figure 1J). The *wag31* K20A mutant also has decreased HADA staining at the old pole, but equivalent staining at the new pole, compared to WT (Figure 1G), which corroborates the elongation data indicating that this mutant is specifically defective in old-pole elongation. The *wag31* K20A strain has similar relative septal HADA intensity as the WT strain (Figure 1H); however, the septal location is shifted slightly toward the old pole (Supplementary Figure S6C), likely because the old pole is elongating more slowly.

The old pole of the *wag31* L34A strain elongates the same as the WT strain, but the new pole has ~76% less elongation (Figure 1J). In the population of *wag31* L34A mutants that we characterized, ~65% had no observable elongation at the new pole, while only 11% of cells in the *wag31* WT population had no new pole elongation in the 1.5 h assay. These assays are performed in unsychronized populations of cells, so the new poles observed are at different stages in their development. Thus, it appears that the Wag31 L34 residue contributes to both initiation and extension of the new pole. The short cell length and slow growth rate (Figures 1B, D,E) are likely due to this defect in new pole elongation, as we did not observe defects in septal HADA staining (Figure 1H; Supplementary Figure S6C).

In the *wag31* F255A mutant, there is an increased relative intensity of HADA at the new pole, and less at the old pole (Figure 1G). The elongation test shows that the *wag31* F255A strain elongates ~47% more than the WT at the new pole, while the old pole elongates 6% less compared to the WT strain (Figure 1J). The HADA relative septal intensity is similar in the *wag31* F255A mutant compared to the WT (Figure 1H), while septal location is shifted toward the old pole, likely due to the increased elongation at the new pole (Supplementary Figure S6C). The increased elongation must be accompanied by septation at a shorter cell length to cause the short cells observed in this mutant (Figure 1E). Our data indicate that the *wag31* F255A mutant is defective in inhibition of new pole synthesis, suggesting that Wag31 plays a role in this function. The data from the K20A, L34A and F255A mutants combined indicates that Wag31 has distinct roles at the two cell poles.

We localized the Wag31 K20A-GFPmut3, Wag31 L34A-GFPmut3, and Wag31 F255A-GFPmut3 in merodiploid strains. Localization of the three mutant proteins is similar to the Wag31 WT at the cell poles (Figure 2C). Localization of the K20A and L34A mutant proteins are slightly dimmer than the WT at the septum (Figure 2D) where new poles are formed during division (Santi et al., 2013). These data indicate that the Wag31 K20A and L34A mutant proteins may be slightly impaired in their ability to adhere to the polar membranes.

### Phosphorylation of Wag31 at T73 Has a minimal impact on peptidoglycan metabolism

Polar cell wall elongation is highly regulated and is known to decrease under stress (Wu et al., 2016; Melzer et al., 2022). In other species, DivIVA homologs involved in cell morphogenesis have been shown to be regulated by phosphorylation (Flärdh, 2003; Kang, 2005; Hempel et al., 2008, 2012; Saalbach et al., 2013; Pompeo et al., 2015; Junker et al., 2018; Passot et al., 2021). Phosphorylation of Wag31 at T73 by PknA (Kang, 2005) has been proposed as a possible means of controlling polar elongation (Jani et al., 2010), as expression of the *wag31* T73E (phospho-mimetic) was observed to increase vancomycin staining of *Msmeg*, while expression of *wag31* T73A (phospho-ablative) decreased staining (Jani et al., 2010).

**Figure 3:**
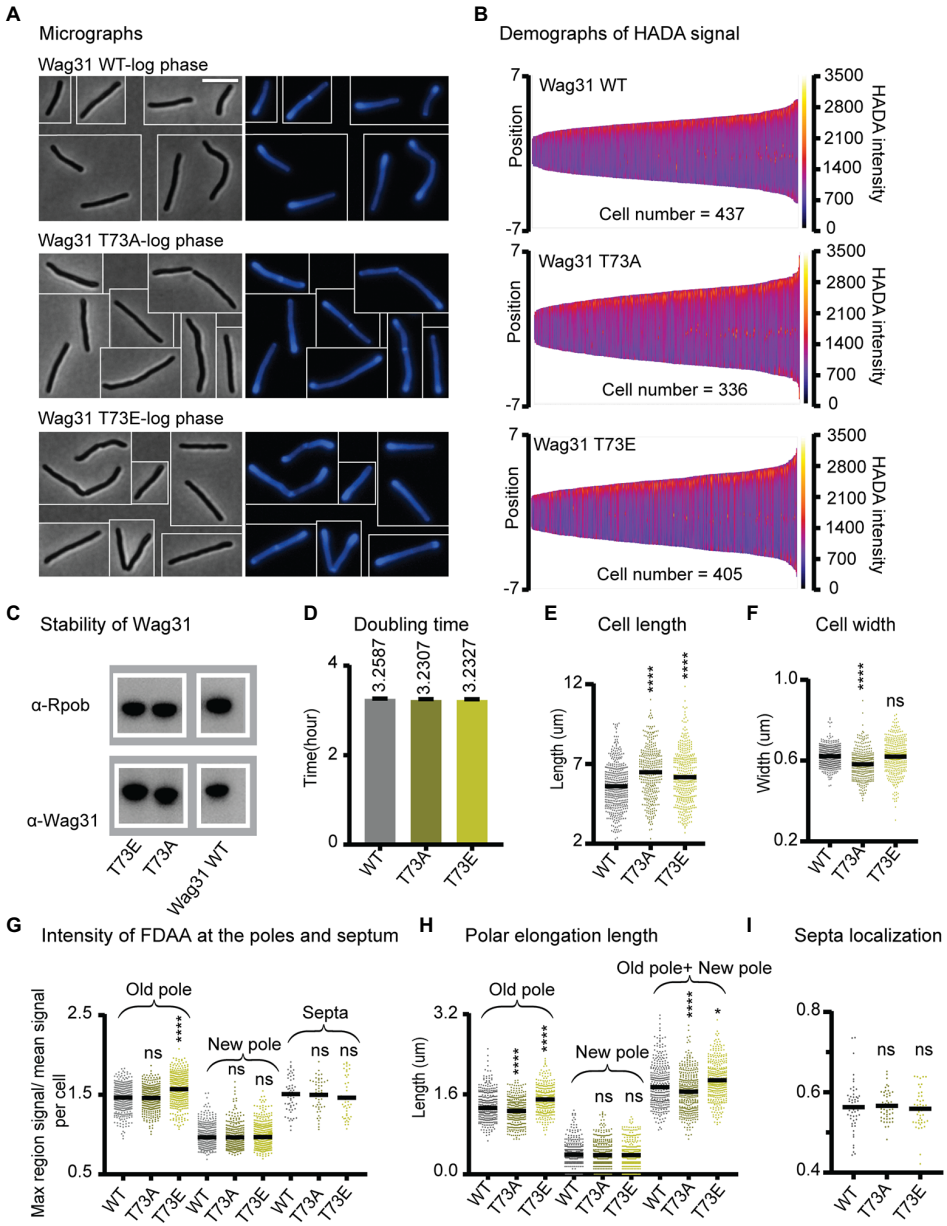
Phosphorylation of T73 on Wag31 subtly affects the cell wall metabolism, but does not affect growth rate. **(A)** Phase (left) and fluorescence (right) images of Msmeg wag31 allele strains stained with HADA. The scale bar is 5 microns, and it applies to all images. **(B)** Demographs of HADA intensity (color scale) across the length of the cell (Y axis) of the wag31 allele strains. The cells were sorted by length, with shortest cells on the left and longest on the right of each demograph. Cells were also pole-sorted according to HADA intensity, such that the brighter pole (presumed to be the old pole) was oriented to the top along the Y axis. At least 100 cells were analyzed from each of three independent biological replicates of each strain. **(C)** Western blots of Wag31 WT, T73A, and T73E proteins in wag31 allele strains using *α*-Wag31 antibody. RpoB serves as a loading control. **(D)** Doubling times of Msmeg cells expressing WT or wag31 mutants. The means (on top of bars) are an average of three biological replicates. Error bars represent SD. **(E)** Cell lengths of the wag31 allele strains. Black bars are at the mean. **(F)** Cell widths of the wag31 allele strains. Black bars are at the mean. **(G)** Relative polar and septal HADA intensity of wag31 allele strains. Relative intensity is equal to the maximum signal at a cell pole, divided by the mean signal of that cell. **(H)** Length of polar elongation in the wag31 allele strains, as measured by the elongation assay method diagrammed in Figure 1I. **(I)** Septal location in wag31 allele strains. Black bar is at the median. ns, *p* > 0.05, ****p = < 0.0001. All *p*-values were calculated by one-way ANOVA, Dunnett’s multiple comparisons test.

To further test this hypothesis, we built the *wag31* T73A and *wag31* T73E strains through allele swapping with the native promoter, in order to control for protein levels, (Figure 3). We found that the Wag31 phospho-mutant proteins are both as stable as the WT (Figure 3C). The mutant strains have similar growth rates compared with the WT (Figure 3D) which indicates that phospho-site T73 does not have a significant role in regulating cell growth. However, we do observe some very subtle effects of these mutations on cell wall metabolism. Both mutants are a little longer (Figure 3E) and the *wag31* T73E strain has slightly more HADA staining at the old pole, while both strains have similar relative HADA signal to the WT at the new pole (Figure 3G). The elongation assay showed that the *wag31* T73E strain elongates ~9% more and the wag31 T73A strain elongates ~8% less than the WT at the old pole. We did not observe significant differences in elongation at the new pole (Figure 3H). The increased cell length (Figure 3E) and slightly decreased in old pole elongation of the *wag31* T73A strain (Figure 3H) suggest that this mutant also activates division at longer cell lengths.

In order to determine whether phosphorylation of Wag31 might regulate cell wall metabolism under stress, we performed HADA staining and microscopy of the same strains in stationary phase (Supplementary Figure S7A). The results show that these phospho-mutants of Wag31 have the same cell length as the WT (Supplementary Figure S7C), indicating that phosphorylation on Wag31 is not required for regulating cell length and polar growth in stationary phase. We found slight differences in HADA staining at the old pole between the mutants and the WT, which are curiously in the opposite direction as in logarithmic phase, with the *wag31* T73A phospho-ablative mutant staining more brightly and the phospho-mimetic mutant staining more dimly (Supplementary Figure S7F). This suggests that phosphorylation on Wag31 may have some minor role in regulating cell wall metabolism in stress, but that it is likely downstream of other regulators. The *wag31* T73A mutant has a higher percentage of cells with active septa in stationary phase (Supplementary Figures S7E,F), indicating that the function of unphosphorylated Wag31 in slowing septation may continue into stationary phase, but that this regulation is not sufficient to affect cell length.

Recent work suggests that Wag31 is a favored substrate of the Serine Threonine Phosphatase PstP, which localizes to the septum during late division (Shamma et al., 2022). This suggests that Wag31 is dephosphorylated at the septum and may primarily be phosphorylated at the poles. It may be that the phosphorylation only accumulates sufficiently to affect peptidoglycan metabolism once the pole has matured into an old pole, since there are no differences in HADA staining between the WT and phospho-mutants at the new pole (Figures 3G,I). In summation, our data with the phospho-mutants indicates that phosphorylation on T73 of Wag31 has only very minor effects on polar growth at the old pole, while the unphosphorylated form may slightly inhibit both polar elongation and septation. However, as these effects are not enough to impact cell growth rates (Figure 3D), we conclude that phosphorylation on T73 is not an important regulator of cell wall metabolism under the conditions tested.

### Wag31 modulates acetyl-CoA carboxylase activity

Wag31 has been shown to interact with AccA3 (Meniche et al., 2014; Xu et al., 2014), a member of the essential ACCase complexes that catalyze the first committed step of the synthesis of the precursors of all fatty acids, mycolic acids, and other methyl-branched lipids (Gago et al., 2011). We hypothesized that this interaction might allow Wag31 to localize and/or regulate the activity of the ACCase complexes, and might therefore affect polar elongation. To test this, we measured ACCase activity in the WT and two of the mutants with defects in polar elongation, *wag31* K20A and *wag31* L34A, as well as two mutants with little effect on polar elongation, the *wag31* T73A and T73E mutants.

**Figure 4:**
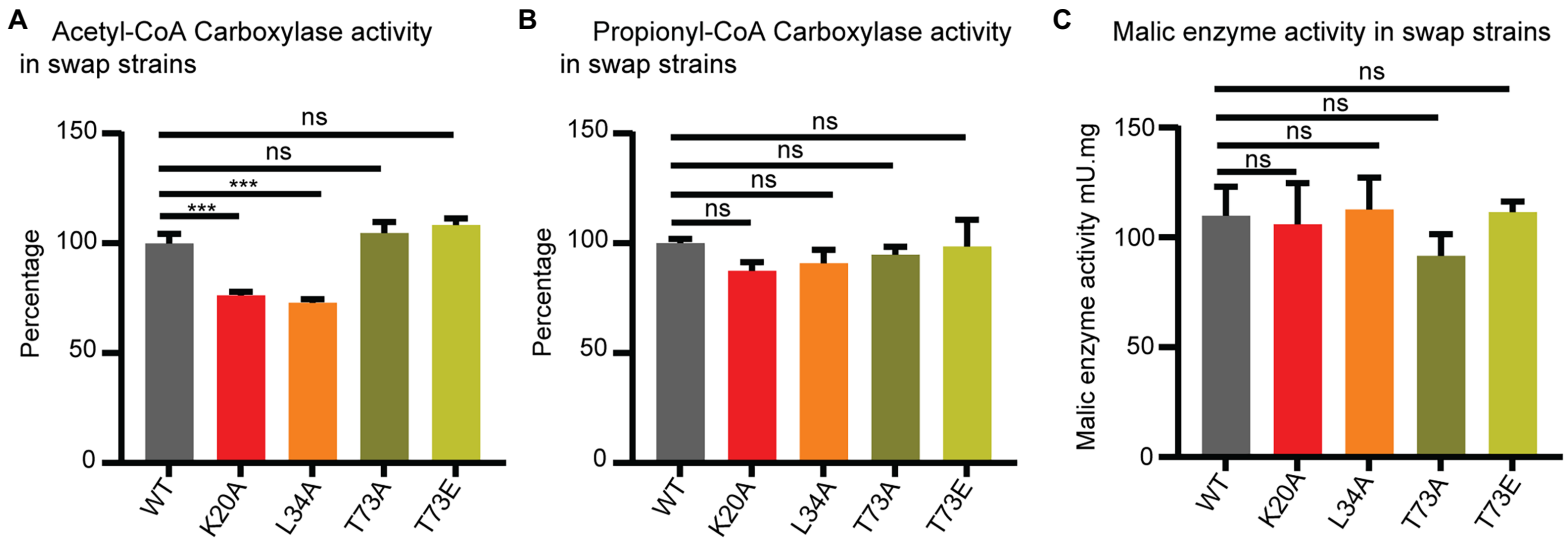
Wag31 modulates ACC activity. ACC **(A)**, PCC **(B)** and malic enzyme **(C)** activity in cell-free extracts of *Msmeg* expressing WT or *wag31* mutants. Results are the means of three independent experiments ± standard deviations (*n* = 3). ns, *p* > 0.05, ***p = <0.0005. All *p*-values were calculated by Tukey’s multiple comparison test.

The ACCase activity was measured by the incorporation of radioactive sodium hydrogen carbonate into the non-volatile fraction of cell extracts with the addition of either acetyl-CoA (to assay ACC activity) or propionyl-CoA (to assay PCC activity) as the enzyme substrates. As shown in Figure 4A, lysates from the *wag31* K20A and *wag31* L34A mutants show a decrease in ACC activity compared with the WT strain, while PCC activity was only slightly modified in the K20A mutant (Figure 4B). As a metabolic control, we also assayed the NADPH dependent malic enzyme activity in the three strains, and found no differences in their levels (Figure 4C), suggesting that the differences in ACC activities was not due to a pleiotropic effect on cell metabolism resulting from the growth defect of the mutants.

The ACC and PCC activities in the cell lysates from the *wag31* T73A and *wag31* T73E strains did not show changes in either of the ACCases or malic enzyme activities.

From these results we conclude that Wag31 impacts the activity of these essential ACCases, either directly or indirectly. The phosphorylation state of Wag31 does not appear to affect the activity of the ACCase enzymes.

In order to assess whether native Wag31 has a direct effect on the activities of ACCases 5 and/or 6, we reconstituted the two enzyme complexes *in vitro* and assayed their activities with their preferred substrates, acetyl-CoA or propionyl-CoA, respectively, and in the presence and absence of Wag31. We found that Wag31 reduces ACCase6 activity ~20% (Supplementary Figure S10A). We also tested the ACCase5 complex, and found that Wag31 does not affect its PCCase activity (Supplementary Figure S10B). These results confirm that at least *in vitro*, native Wag31 modulates ACCase6 activity and suggests that the Wag31 K20A and Wag31 L34A mutants might have a stronger modulatory effect *in vivo* compared with the wild type protein.

### Wag31 does not regulate polar elongation by controlling the localization of cell wall precursor enzymes to the IMD

Recent work suggests that localization of the enzymes that synthesize cell wall precursors to the subpolar Intracellular Membrane Domain (IMD) may be critical in restricting elongation to the pole (Hayashi et al., 2016; Melzer et al., 2022). Depletion of Wag31 has been shown to delocalize the IMD (García-Heredia et al., 2021), though it is not known if Wag31 regulates the IMD, or if the localization of IMD-associated enzymes is merely dependent on an intact cell pole.

**Figure 5:**
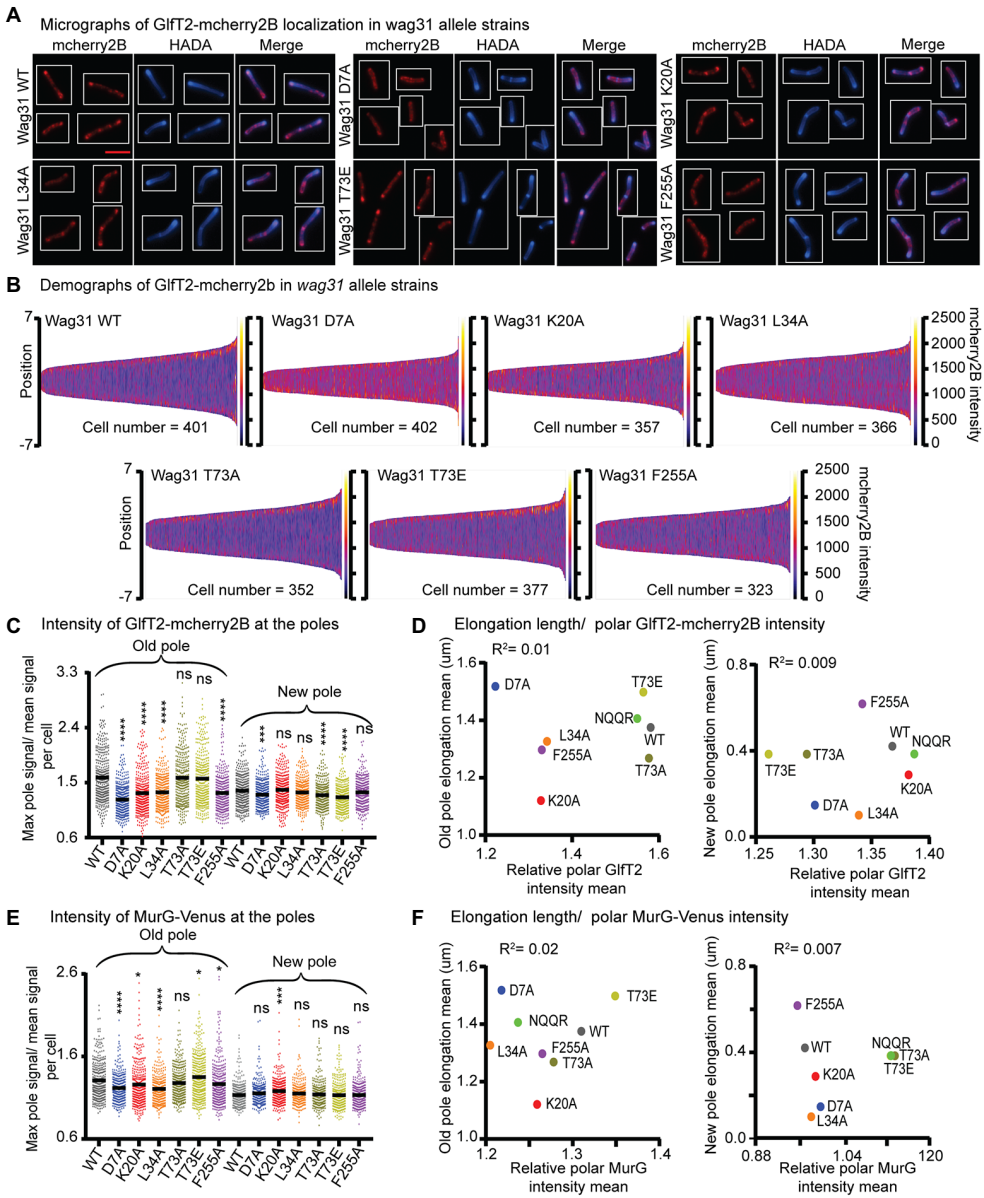
Localization of IMD proteins does not determine changes in polar growth. **(A)** Micrographs of wag31 allele strains expressing GlfT2-mcherry2B and stained with HADA. The scale bar is 5 microns, and it applies to all images. **(B)** Demographs of GlfT2-mcherry2B intensity across the length of each cell (Y axis of each plot, with intensity indicated by color – scale to the right of each plot), arranged by cell length (along the X axis). At least 100 cells were analyzed for each of three biological replicate cultures. **(C)** Relative polar intensity of GlfT2-mcherry2B in the Wag31 WT and Wag31 mutants at the old pole and the new pole. Relative intensity is equal to the maximum signal at a cell pole, divided by the mean signal of that cell. **(D)** Correlation between relative polar intensity of GlfT2-mcherry2B and elongation length (Figure 1J) at the poles. The eight mutants shown as colored dots. **(E)** Relative polar intensity of MurG-Venus in the Wag31 WT and Wag31 mutants at the old pole and the new pole. **(F)** Correlation between relative polar intensity of MurG-Venus and elongation length at the poles. ns, *p* > 0.05, *p = <0.05, ***p = <0.0005, ****p = <0.0001. *p*-values were calculated by one-way ANOVA, Dunnett’s multiple comparisons test.

To probe whether Wag31 regulates IMD structure, we localized both GlfT2-mcherry2B and MurG-Venus in a subset of *wag31* alanine-mutant strains (Figure 5; Supplementary Figure S3). GlfT2 is the last cytoplasmic galactan enzyme in arabinogalactan synthesis (Beláňová et al., 2008), associates with the IMD, and localizes in the typical pattern of IMD-associated proteins (Hayashi et al., 2016) in the subpolar region and spottily along the lateral walls. MurG is the final cytoplasmic enzyme in peptidoglycan precursor synthesis, has a similar localization pattern as GlfT2, (Hayashi et al., 2016) and is found in both cytoplasmic and IMD membrane fractions (García-Heredia et al., 2021).

Our microscopy data show that localization of GlfT2-mcherry2B at the old pole is significantly reduced in the Wag31 K20A, D7A, L34A and F255A mutants compared to the WT, while it was unchanged in the rest of the mutants (Figures 5A–C; Supplementary Figure S3I). At the new pole, localization of GlfT2-mcherry2B is largely unaffected, with modest defects in localization in the *wag31* D7A, T73A, and T73E strains (Figures 5A–C; Supplementary Figure S3I). Localization of MurG-Venus is slightly decreased at the old pole in the Wag31 D7A and L34A mutants compared to the WT (Figure 5E; Supplementary Figure S3J), while it is unchanged in all mutants at the new pole (Figure 5E; Supplementary Figure S3J).

To test the hypothesis that the localization of IMD proteins helps determine polar growth (Mohammadi et al., 2007; Hayashi et al., 2018), we plotted the polar IMD signal of GlfT2 and MurG against the polar elongation metric for each *wag31* allele strain. Across the mutants tested, polar elongation does not correlate either with GlfT2 (Figure 5D) or with MurG localization signals at the poles (Figure 5F). These data indicate that Wag31 may regulate the localization of IMD proteins, probably indirectly and largely at the old pole, but that this regulation does not directly control polar elongation.

### Wag31 does not regulate the cell wall during logarithmic phase by regulation of FtsI through residues NSD46-48

**Figure 6:**
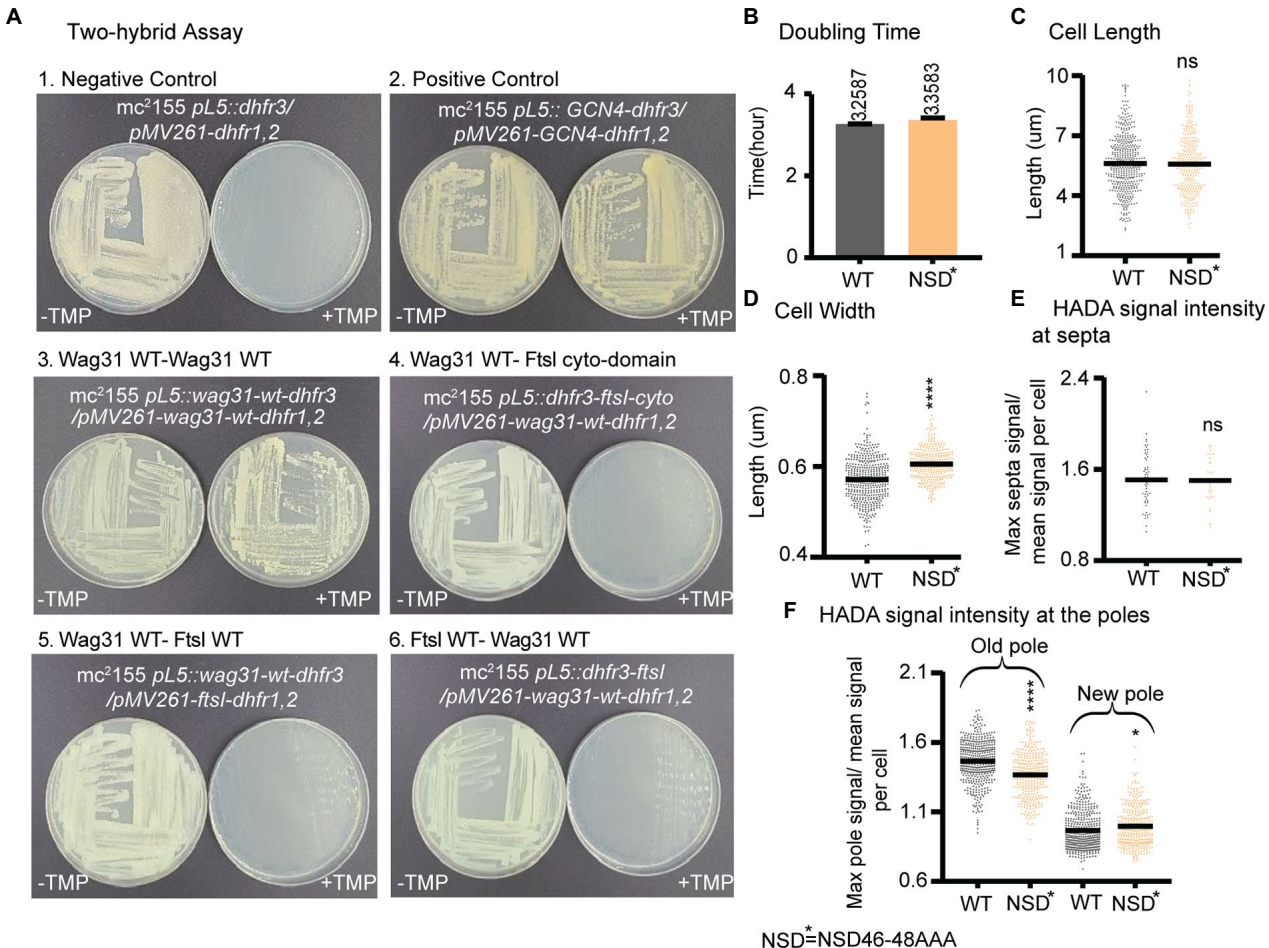
Wag31 does not regulate FtsI in logarithmic phase through residues NSD46-48. **(A)** Representative plates from the Mycobacterial Protein Fragment Complementation two-hybrid assay. Indicated strains were struck out on plates with and without trimethoprim (+TMP/ -TMP). **(B)** Doubling times of *Msmeg* cells expressing WT or *wag31* NSD46-8AAA mutant. **(C)** Cell lengths and **(D)** Cell width of the *wag31* NSD46-8AAA strain. Black bars are at the mean. **(E)** Relative septal and polar HADA **(F)** intensity of Wag31 WT and Wag31 NSD46-8AAA. ns, p > 0.05, *p = <0.05, ***p = <0.0005, ****p = <0.0001. *p*-values were calculated by unpaired *t*-test.

Previous work showed that Wag31 interacts with the septal PBP3, FtsI under oxidative stress, and regulates FtsI stability through Wag31 residues 46–48 (NSD) (Mukherjee et al., 2009). FtsI is a peptidoglycan synthase with transpeptidation activity which is recruited to the septum by interacting with FtsW (Datta et al., 2006). We tested the interaction between Wag31_Mtb_ and FtsI_Mtb_ with the Mycobacterial Protein Fragment Complementation two-hybrid assay (Singh et al., 2006). We used constructs with both the full length and cytoplasmic domain of FtsI. However, we were not able to confirm the Wag31-FtsI interaction in these conditions using this method (Figure 6A).

Next, we made an *Msmeg* allele swap strain with a *wag31* ∆46–48 mutant, as per (Mukherjee et al., 2009) and found this mutant protein was unstable, while a *wag31* NSD46-48AAA mutant was stable (Supplementary Figure S2). So, we used a *wag31* NSD46-48AAA swap strain to test whether the Wag31-FtsI interaction, previously demonstrated in in oxidative stress (Mukherjee et al., 2009), has any role in regulating cell wall metabolism in logarithmic phase (Figure 6). We found that the *wag31* NSD46-48AAA mutant has a normal cell length and growth rate (Figures 6B,C). We conclude that residues NSD46-48 of Wag31 do not affect FtsI activity in logarithmic phase (Figures 6E,F). Because we were not able to observe a protein interaction in the two-hybrid assay, we think it likely that Wag31 affects septation through another septal protein during logarithmic phase.

## Discussion

Wag31’s molecular function in controlling cell wall metabolism has remained poorly understood. Homology with other systems predicts that Wag31 orthologs might recruit and regulate cell wall enzymes at the pole (Marston and Errington, 1999; Sieger and Bramkamp, 2015). However, it is not clear how the hetero-oligomeric interactions that have been described for Wag31 – AccA3 (Mukherjee et al., 2009) and FtsI (Mukherjee et al., 2009) – connect to Wag31’s role in restricting peptidoglycan metabolism to the poles. In this work, we built and phenotyped a number of *wag31* point mutant strains in an attempt to genetically separate Wag31’s functional roles.

**Figure 7:**
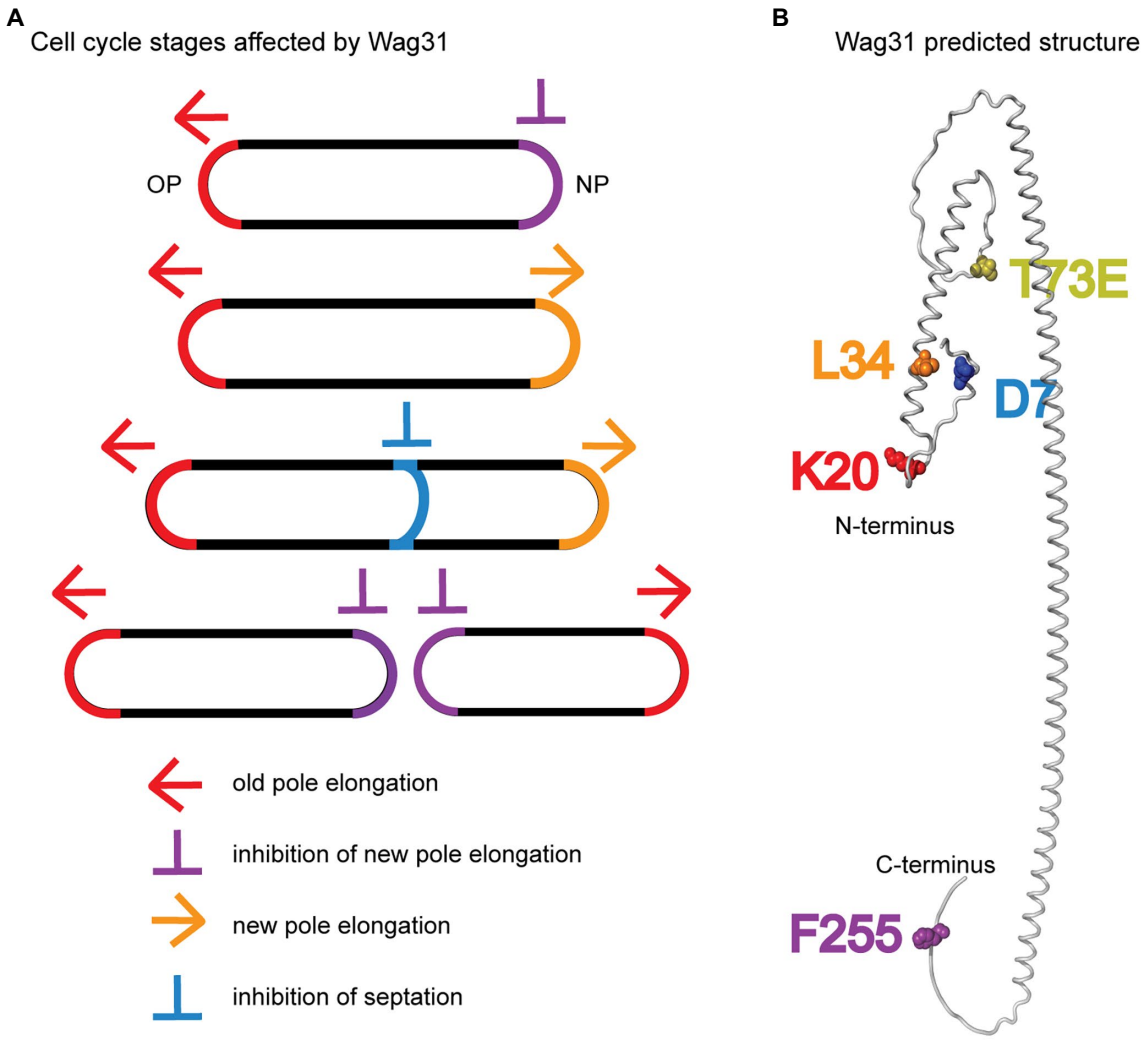
Models. **(A)** Multiple stages of the cell cycle affected by Wag31 are highlighted. **(B)** Alphafold2 predicted structure (Jumper et al., 2021) of a Wag31 monomer, with residues of interest highlighted.

We characterized a handful of *wag31* point mutants which together indicate that Wag31 has distinct functions in polar elongation (Figures 7A,B). We found that the *wag31* K20A mutant is specifically more defective in old pole elongation while the *wag31* L34A mutant is specifically more defective in new pole elongation (Figure 1). These results indicate that Wag31 plays different roles at the new and old poles; it may have a distinct homo-oligomeric conformations at the two poles, or may form different hetero-oligomeric regulatory interactions. Both of these mutants exhibited slower growth rates, and decreased ACC activity compared to the WT (Figures 1D, 4A). The effect of Wag31 mutants on ACC activity suggests that the native conformation of Wag31 helps keep optimal levels of the ACCase complex working in the initial steps of lipid synthesis, contributing to preserve the integrity of the cell membranes (Gago et al., 2011). Modulation of the ACCase complex activities may occur through the Wag31-AccA3 interaction which has been described before (Meniche et al., 2014; Xu et al., 2014). Our data do not indicate whether Wag31 controls polar peptidoglycan synthesis by a separate pathway than that by which it regulates ACCase activity, or whether peptidoglycan synthesis is controlled in coordination with lipid synthesis. The transglycosylases that catalyze incorporation of new cell wall material are all strongly associated with the cytoplasmic membrane (Hayashi et al., 2016; Meeske et al., 2016; Arora et al., 2018), and the lipidII peptidoglycan precursor is made and transported through the membrane (Gee et al., 2012; Sham et al., 2014; Garcia-Heredia et al., 2019); thus, peptidoglycan synthesis could be regulated by the composition or protein-occupation status of the membrane, which could be regulated by Wag31’s effect on the ACCase complex.

We also found that the *wag31* F255A mutant had increased polar elongation at the new pole (Figure 1). This suggests that Wag31 has a distinct role in regulating new pole elongation. LamA is a membrane protein that has previously been shown to inhibit new pole synthesis in *Msmeg* and which thereby contributes to cell asymmetry and heterogeneity (Rego et al., 2017). Wag31, through its C-terminus, may either work separately to inhibit new pole elongation, or may work in a pathway with LamA.

Our data suggest that Wag31 may have a minor role in controlling the timing or rate of septation. Wag31 had previously been shown to localize to the cell division site after FtsZ has left (Gupta et al., 2022) but before septation is completed (Santi et al., 2013), and overexpression of Wag31-GFP was shown to inhibit and mis-localize septa (Nguyen et al., 2007). Previous work showed that the Wag31-FtsI interaction protects FtsI from intra-membrane proteases under oxidative stress through Wag31 residues 46–48 (NSD) (Mukherjee et al., 2009). Our data show that these residues do not affect cell division in exponential phase (Figure 6). However, we do find evidence of mis-regulation of septation in the *wag31* D7A strain. So, the Wag31 D7 residue could possibly establish a different interaction with FtsI, or with other septal factors (Wu et al., 2018). The phospho-site T73 on Wag31 may also have a role in regulating septation: we observed that the *wag31* T73A phospho-ablative mutant has slower elongation but longer cell length in logarithmic phase (Figures 3E,G,H), which suggests that it has delayed cell division. Delayed cell division of the *wag31* T73A is also apparent in stationary phase stress, as there are more cells with active peptidoglycan metabolism at the septum than in the WT strain (Supplementary Figure S7E).

Most of the mutants we characterized show a defect in GlfT2-mcherry2B and MurG-Venus localization at the old pole, but not new pole. The localization of GlfT2 and MurG at the poles did not correlate with polar elongation defects (Figure 5; Supplementary Figure S3), indicating that polar localization of the IMD proteins is not a key determinant of polar growth. These data suggest that Wag31 is at best an indirect regulator of the IMD, and that regulation of the localization of IMD proteins is likely not Wag31’s essential function.

In this work, we define new roles for Wag31 in regulating both septation and the inhibition of the new pole, and identify residues that have dominant roles in Wag31’s different functions (Figure 7). We show that Wag31 can modulate the activity of the ACCase complex and therefore may have a role in maintaining lipid homeostasis in mycobacteria (Figure 4). We also show that phosphorylation of Wag31 at T73 has very little effect on cell wall metabolism (Figure 3; Supplementary Figure S7). In addition, we show that Wag31 does not affect FtsI in logarithmic phase through the same residues used to regulate FtsI in stress (Mukherjee et al., 2009).

## Data availability statement

The original contributions presented in the study are included in the article/Supplementary material, further inquiries can be directed to the corresponding author.

## Author contributions

NH and CB were responsible for conceptualization, validation, and writing the first draft of the manuscript. HG, LD, and DE wrote the sections of the manuscript related to the ACCases. CB supervised the work together with HG and LD. NH, CB, HG, and LD contributed to reviewing and editing the manuscript. AE aided in data processing. NH, DE, and PP were responsible for formal analysis of the data. NH, SQ, and DE conducted the experimental work of the investigation. NH carried out all the microscopical visualization and data presentation. NH was responsible for data curation and methodology. All authors contributed to the article and approved the submitted version.

## Funding

This work was supported by National Institutes of Health (NIH) grant R01AI148917 to CB.

## Conflict of interest

The authors declare that the research was conducted in the absence of any commercial or financial relationships that could be construed as a potential conflict of interest.

## Publisher’s note

All claims expressed in this article are solely those of the authors and do not necessarily represent those of their affiliated organizations, or those of the publisher, the editors and the reviewers. Any product that may be evaluated in this article, or claim that may be made by its manufacturer, is not guaranteed or endorsed by the publisher.
