# Supplementary material for: Polar protein Wag31 both activates and inhibits cell wall metabolism at the poles and septum
Source: Front Microbiol. 2023 Jan 12;13:1085918. doi: 10.3389/fmicb.2022.1085918 (PMC9878328; doi:10.3389/fmicb.2022.1085918)
Supplement: Supplementary file 1 [file Data_Sheet_1.PDF]

```
%Code. S1. This code is providing the intensities of old and new poles
(pole1, pole2). Also, it is calculating the mean intensity of each
cell.

filn='filename'; % Insert your file name here
importfiln=append(filn,'.xlsx');
resultfiln=append(filn,'-RSL.xlsx');
transformedfiln=append(filn,'-TRNS.xlsx');
data=xlsread(importfiln,'B:C');
[n,r]=size(data);
count=1;
length=1;
for i=1:n
    if data(i,1)<1
        newdata(2*count-1,length)=data(i,1);
        newdata(2*count,length)=data(i,2);
        length=length+1;
    else
        newdata(2*count-1,length)=data(i,1);
        newdata(2*count,length)=data(i,2);
        lengthlog(count,1)=count;
        lengthlog(count,2)=length;
        count=count+1;
        length=1;
    end
end
count=count-1;
for k=1:count
    pole1=0;
    pole2=0;
    for l=1:lengthlog(k,2)
        if newdata(2*k-1,l)>=0 && newdata(2*k-1,l)<=0.15
            pole1=[pole1,newdata(2*k,l)];
        end
        if newdata(2*k-1,l)>=0.85 && newdata(2*k-1,l)<=1.0
            pole2=[pole2,newdata(2*k,l)];
        end
        Intensvec(l)=newdata(2*k,l);
    end
    finaldata(k,1)=max(pole1);
    finaldata(k,2)=max(pole2);
    finaldata(k,3)=mean(Intensvec);
    clear pole1 pole2 septa dimest Intensvec
end
xlswrite(resultfiln,finaldata)
xlswrite(transformedfiln,newdata)
```

```
%%Code. S2. This code is providing the septal intensity and its
location in each cell.

filn='filename'; % Insert your file name here
importfiln=append(filn,'.xlsx');
resultfiln=append(filn,'-SeptRSL.xlsx');
transformedfiln=append(filn,'-TRNS.xlsx');
data=xlsread(importfiln,'B:C');
[n,r]=size(data);
count=1;
length=1;
limit=50;
for i=1:n
    if data(i,1)<1
        newdata(2*count-1,length)=data(i,1);
        newdata(2*count,length)=data(i,2);
        length=length+1;
    else
        newdata(2*count-1,length)=data(i,1);
        newdata(2*count,length)=data(i,2);
        lengthlog(count,1)=count;
        lengthlog(count,2)=length;
        count=count+1;
        length=1;
    end
end
count=count-1;
for k=1:count
    septa=0;
    septaloc=0;
    for l=1:lengthlog(k,2)
        if newdata(2*k-1,l)>=0.3 && newdata(2*k-1,l)<=0.8
            septa=[septa,newdata(2*k,l)];
            septaloc=[septaloc,newdata(2*k-1,l)];
        end
    end
    [nn,rr]=size(septa);
    peaknumbers=0;
    for ii=2:rr-1
        if (septa(ii+1)-septa(ii))>=limit
            dimest=septa(ii);
            dimestloc=septaloc(ii);
            peaknumbers(1)=septa(ii+1);
            pplace=ii+1;
            for kk=pplace:rr-1
                if (septa(kk+1)-dimest)>=limit
                    peaknumbers=[peaknumbers,septa(kk+1)];
                else
                    break
                end
```

```
            end
            break
        end
    end
    [row,col]=size(peaknumbers);
    if dimest==0
        finaldata(k,1)=0;
        finaldata(k,2)=0;
    end
    if col<3
        finaldata(k,1)=0;
        finaldata(k,2)=0;
    else
        finaldata(k,1)=max(peaknumbers);
        xx=max(peaknumbers);
        location=find(septa==xx,1);
        finaldata(k,2)=septaloc(location);
    end
    for iii=1:size(peaknumbers,2)
        peakdata(k,iii)=peaknumbers(iii);
    end
    clear septa septaloc peaknumbers dimestloc dimest
end
xlswrite(resultfiln,finaldata)
```
