# Supplementary material for: Polar protein Wag31 both activates and inhibits cell wall metabolism at the poles and septum
Source: Front Microbiol. 2023 Jan 12;13:1085918. doi: 10.3389/fmicb.2022.1085918 (PMC9878328; doi:10.3389/fmicb.2022.1085918)
Supplement: Supplementary file 2 [file Data_Sheet_2.pdf]

A.Multiple sequence alignment of DivIVA proteins across Actinobacteria and Firmicutes phylum

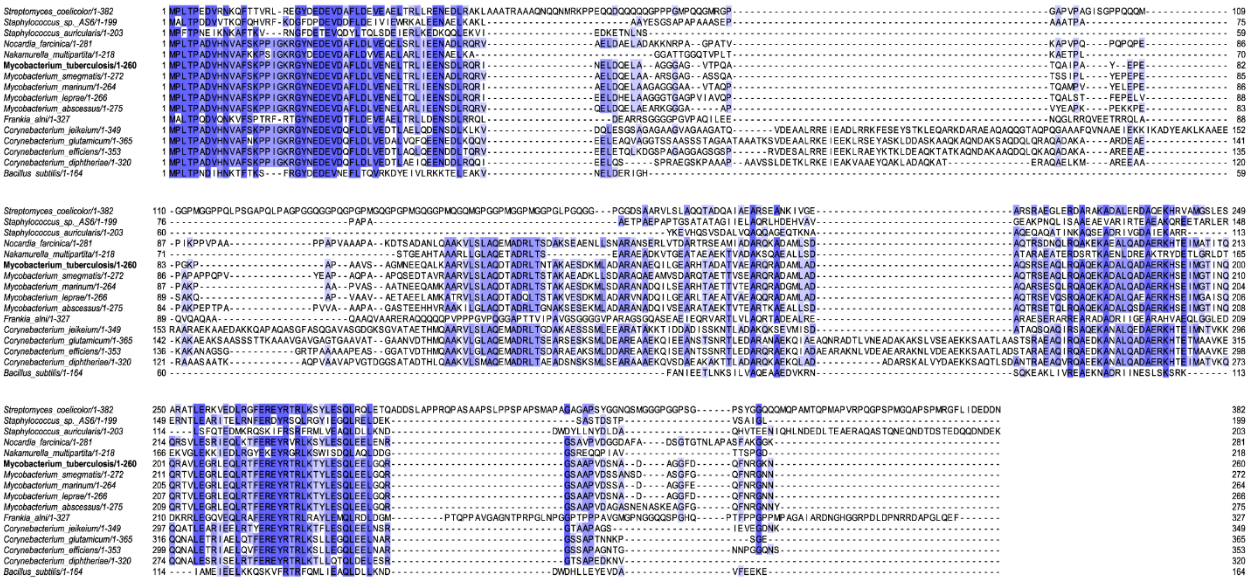

Percentage agreement	Colour
> 80%	blue
> 60%	lavendar
> 40%	light lavendar
<= 40%	White

Supplemental Figure 1. Wag31 vs. DivIVA. (A) Multiple sequence alignment of DivIVA proteins across Actinobacteria and Firmicutes phyla using Clustal Omega program (Goujon *et al.*, 2010; Sievers *et al.*, 2011; McWilliam *et al.*, 2013) and visualized with Jalview version 2(Waterhouse *et al.*, 2009; Troshin *et al.*, 2011).

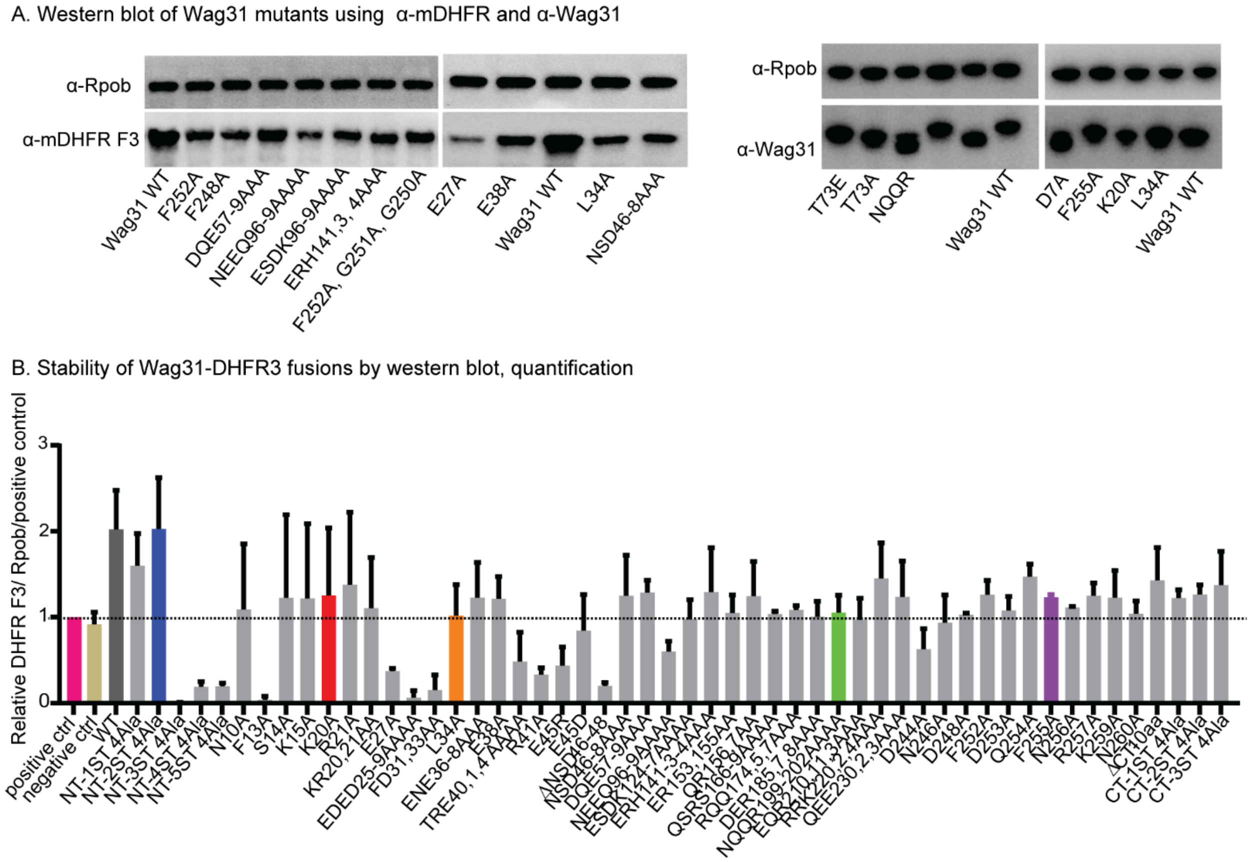

Supplemental Figure 2. Stability of Wag31 mutants. Stability of Wag31 mutant proteins was tested in the context of the Mycobacterial Protein Fragment Complementation Assay (Singh *et al.*, 2006). In this assay, WT *wag31* was fused to the N-terminus (Fragments 1,2) of mDHFR, mutant versions of w*ag31* were fused to the C-terminus (Fragment 3) of mDHFR. The interaction of the mDHFR fragments provides trimethoprim resistance, which is an indication of protein-protein interaction. Here we used western blots against Fragment 3 of mDHFR to assess the stability of the Wag31 mutant proteins. (A) Representative western blots of WT and mutants of Wag31-DHFR3 using α-mDHFR antibody (left), Western blots of Wag31 WT and mutant proteins in wag31 allele strains using α-Wag31 antibody (right). RpoB serves as a loading control. (B) Normalized quantification of western blot (as in B) band intensities: ratio of ((Wag31-allele/ matched RpoB)/(Wag31 WT/ matched RpoB))/(DHFR-GCN4/ matched RpoB). Values are the mean of western blots from two independent cultures. Error bars represent SD. The dotted line shows the protein level of the DHFR positive control.

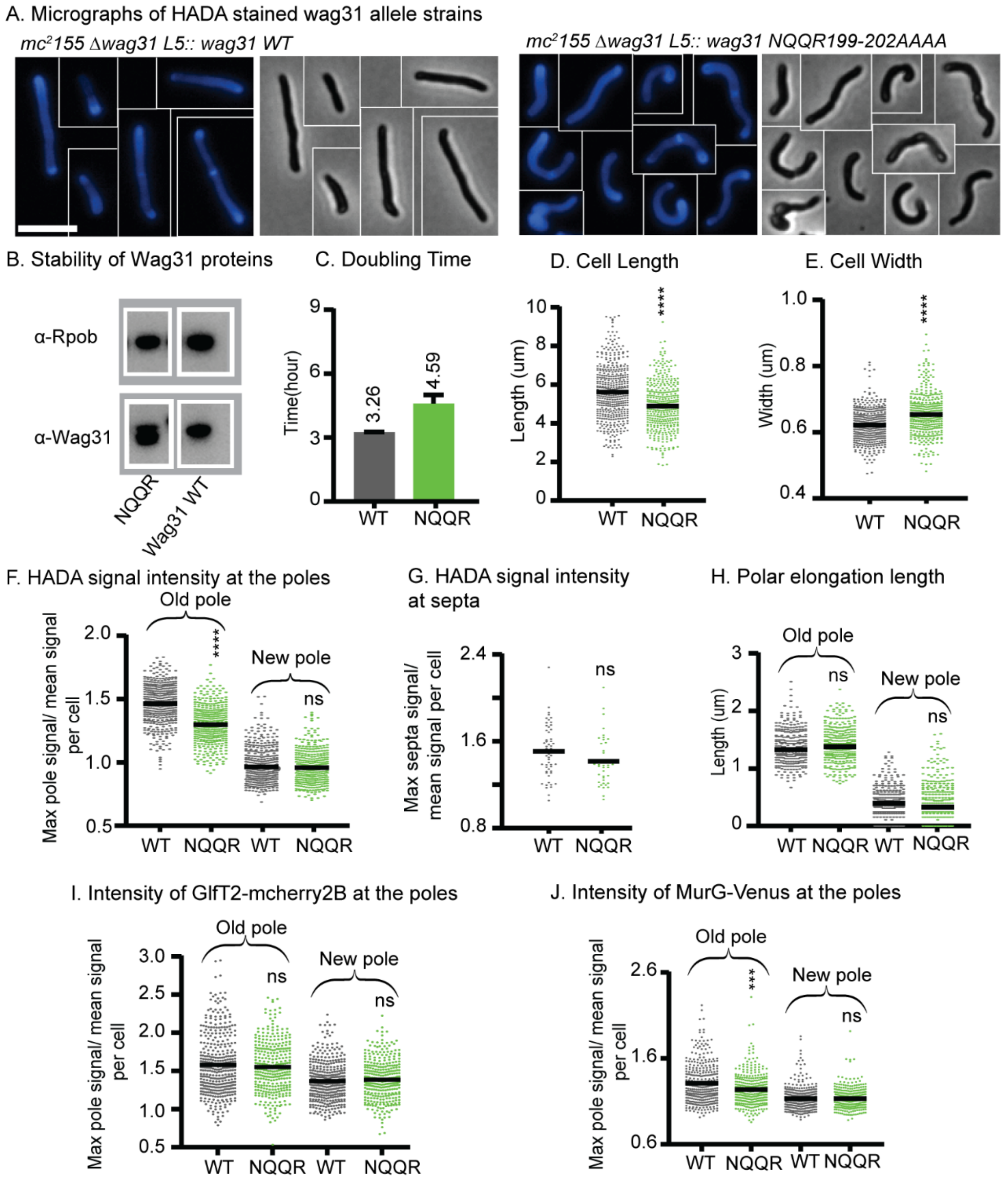

Supplemental Figure 3. The formation of rod-shape morphology is affected by NQQR199-202 residues. (A) Fluorescence (left) and phase (right) images of *Msmeg wag31* allele strains stained with HADA. The scale bar is 5 microns, and it applies to all images. (B) Western blots of Wag31 NQQR199-202AAA and Wag31 WT proteins in *wag31* allele strains using α-Wag31 antibody. RpoB serves as a loading control. (C) Doubling times of *Msmeg* cells expressing WT or *wag31* NQQR199-202AAAA mutant. The means (on top of bars) are an average of three biological replicates. Error bars represent SD. (D) Cell length of the *wag31* NOOR199-202AAAA and *wag31* WT strains. Black bars are at the mean. (E) Cell width of the *wag31* allele strains. Black bars are at the mean. (F) Relative polar and septal (G) HADA intensity of *wag31* allele strains. (H) Length of polar elongation in the *wag31* allele strains. Black bar is at the

median. (I) Relative polar intensity of GlfT2-mcherry2B in the Wag31 WT and Wag31 NOOR199-202AAAA mutants at the old pole and the new pole. (J) Relative polar intensity of MurG-Venus in the Wag31 WT and Wag31 NOOR199-202AAAA at the old pole and the new pole. ns, P >0.05, *, P =< 0.05, ***, P=< 0.0005, ****, P= <0.0001. *P*-values were calculated by the unpaired t-test.

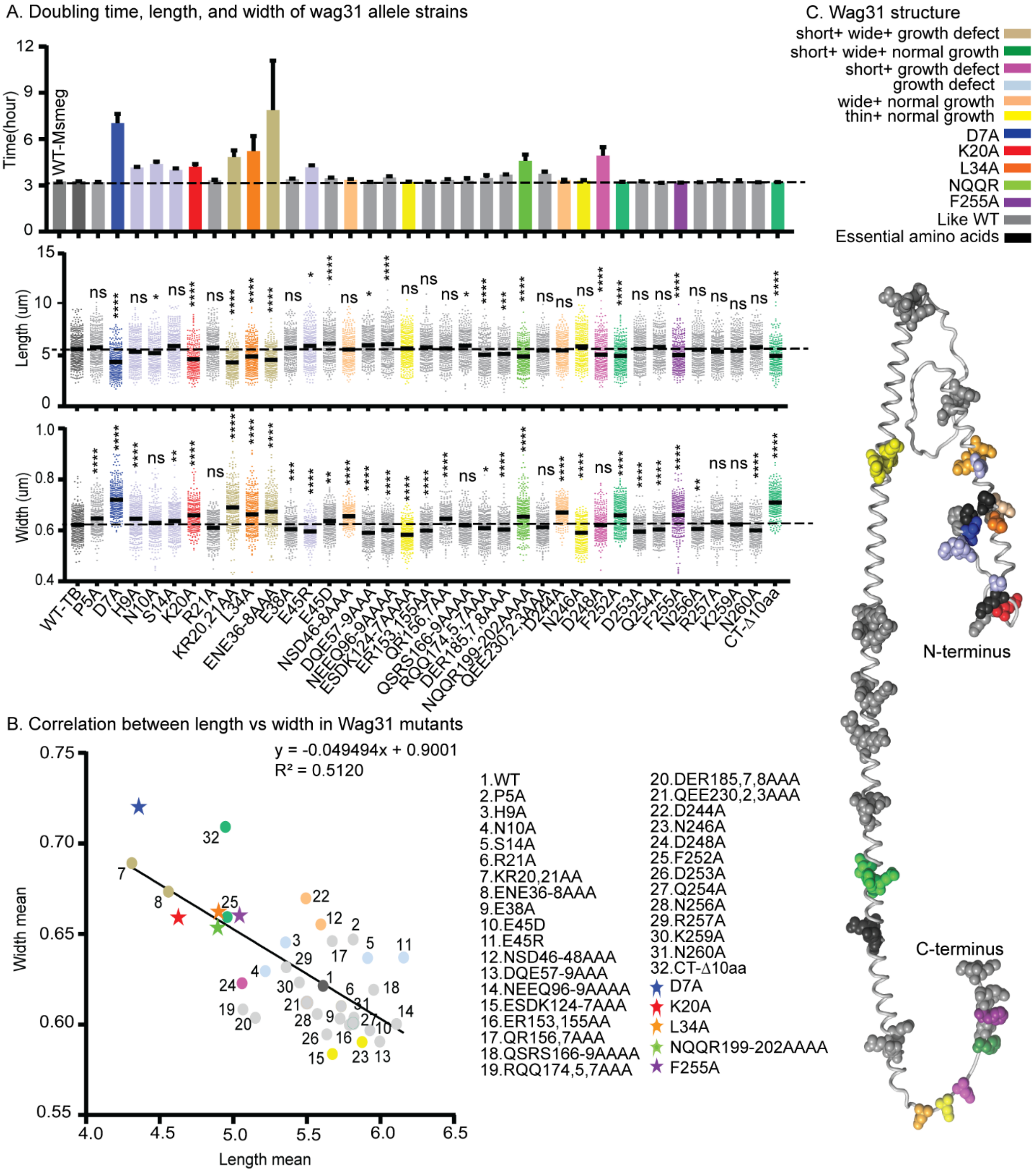

Supplemental Figure 4. Gross cell phenotypes. (A) Doubling time (top), cell length (middle) and cell width (bottom) in *wag31* allele strains. Cells with a mean at least 10% less than the WT

mean, and with a width at least 5% more than the mean of the WT strain, are considered short and wide, respectively. ns, P >0.05, *, P =< 0.05, **, P=< 0.005, ***, P=< 0.0005, ****, P= <0.0001. *P*-values were calculated by one-way ANOVA, Dunnett's multiple comparisons test. (B) Mean length of *wag31* allele strains plotted as a function of their mean width. Black line is a linear fit. (C) Residues mutated in the alanine scanning mutagenesis are shown as spheres on the Alphafold2 predicted structure (Jumper *et al.*, 2021) of Wag31. Residues are colored according to phenotype categories in (A). Essential residues (black) are the mutants that were unable to replace the *wag31* WT allele.

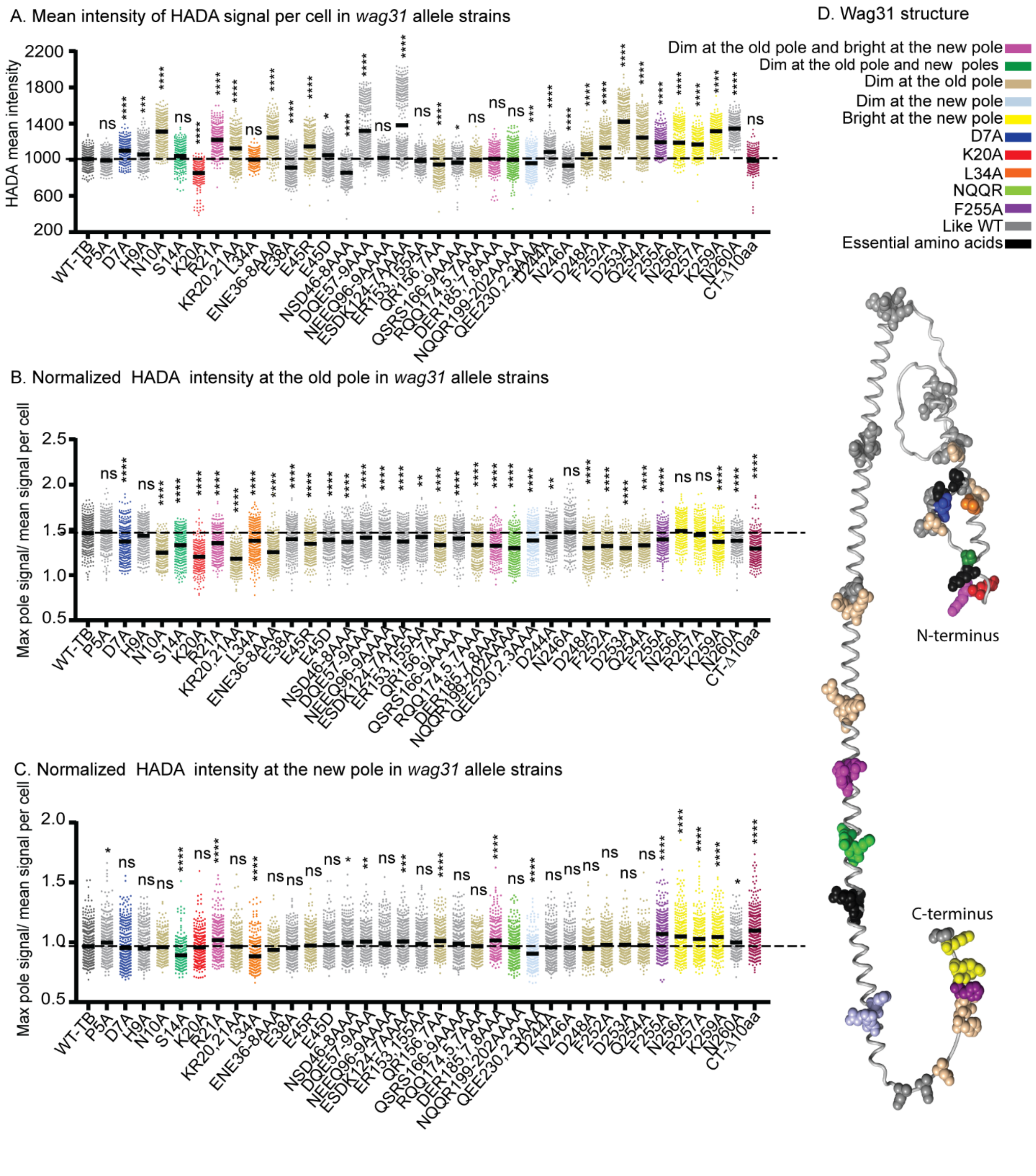

Supplemental Figure 5. Polar peptidoglycan metabolism. (A) Mean intensity of HADA signal per cell in *wag31* allele strains. (B), (C) Relative HADA signal at the old and new poles, normalized to the cell mean. Black bars are at the mean. Cells with polar intensity at least 7% less than the mean of the *wag31* WT strain are considered dim at the old pole. Cells with polar intensity at least 5% more than the mean of the *wag31* WT strain are considered bright at the new pole. Cells with polar intensity at least 5% less than the mean of the *wag31* WT strain are considered dim at the new pole. (D) Residues mutated in the alanine scanning mutagenesis are shown as spheres on the Alphafold2 predicted structure of Wag31. Residues are colored according to phenotype categories in A, B, C. ns, P >0.05, *, P =< 0.05, **, P=< 0.005, ***, P=< 0.0005, ****, P= <0.0001. *P*-values were calculated by one-way ANOVA, Dunnett's multiple comparisons test.

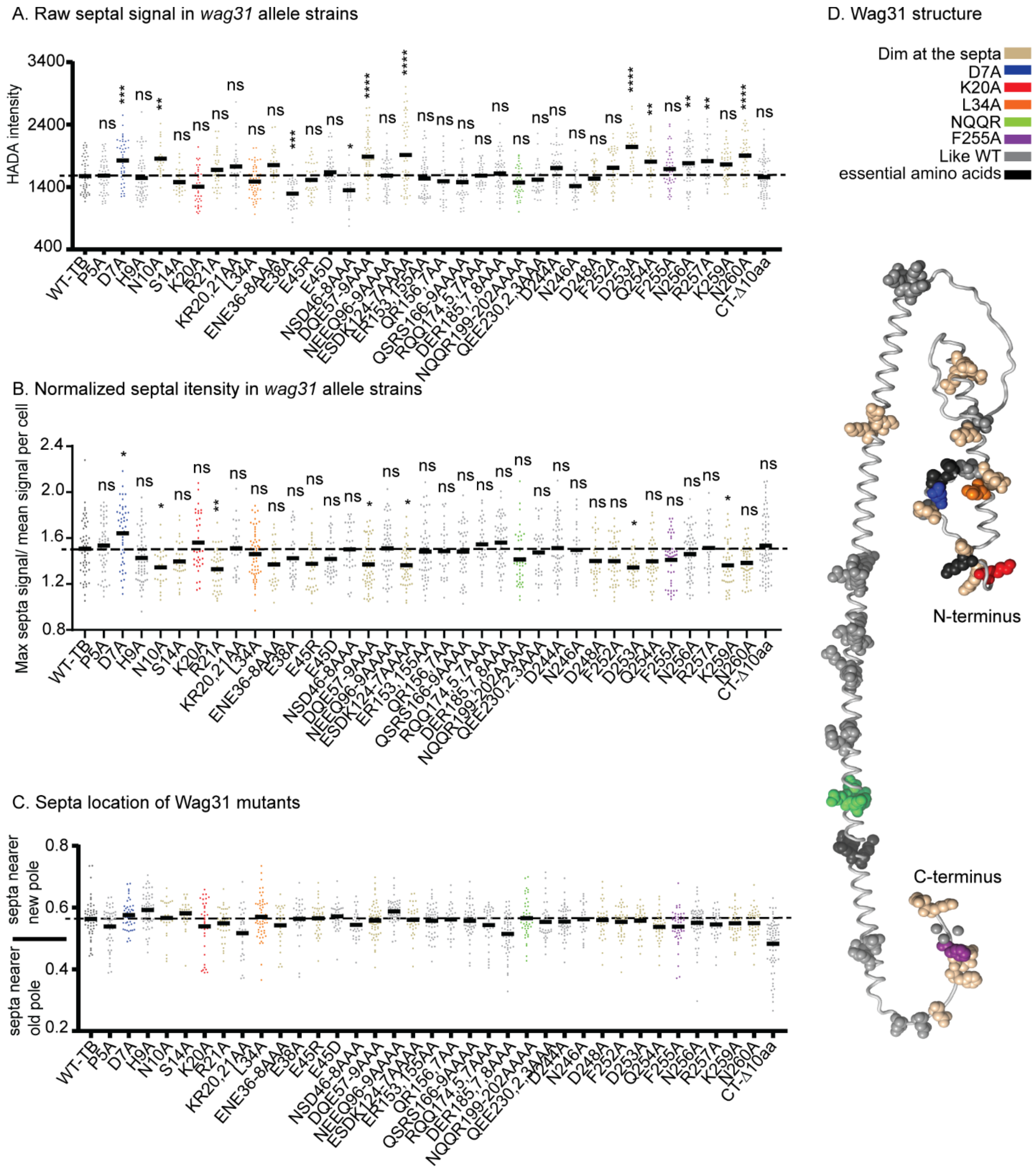

Supplemental Figure 6. Septal peptidoglycan metabolism. (A) Raw and, (B) relative septal HADA intensity of *wag31* allele strains. Black bars are at the mean. (C) Septal location in *wag31* allele strains. (D) Residues mutated in the alanine scanning mutagenesis are shown as spheres on the Alphafold2 predicted structure of Wag31. Residues that have at least 7% less normalized septal intensity than the WT are defined as dim. ns, P >0.05, *, P =< 0.05, **, P=< 0.005, ***, P=< 0.0005, ****, P= <0.0001. *P*-values were calculated by one-way ANOVA, Dunnett's multiple comparisons test.

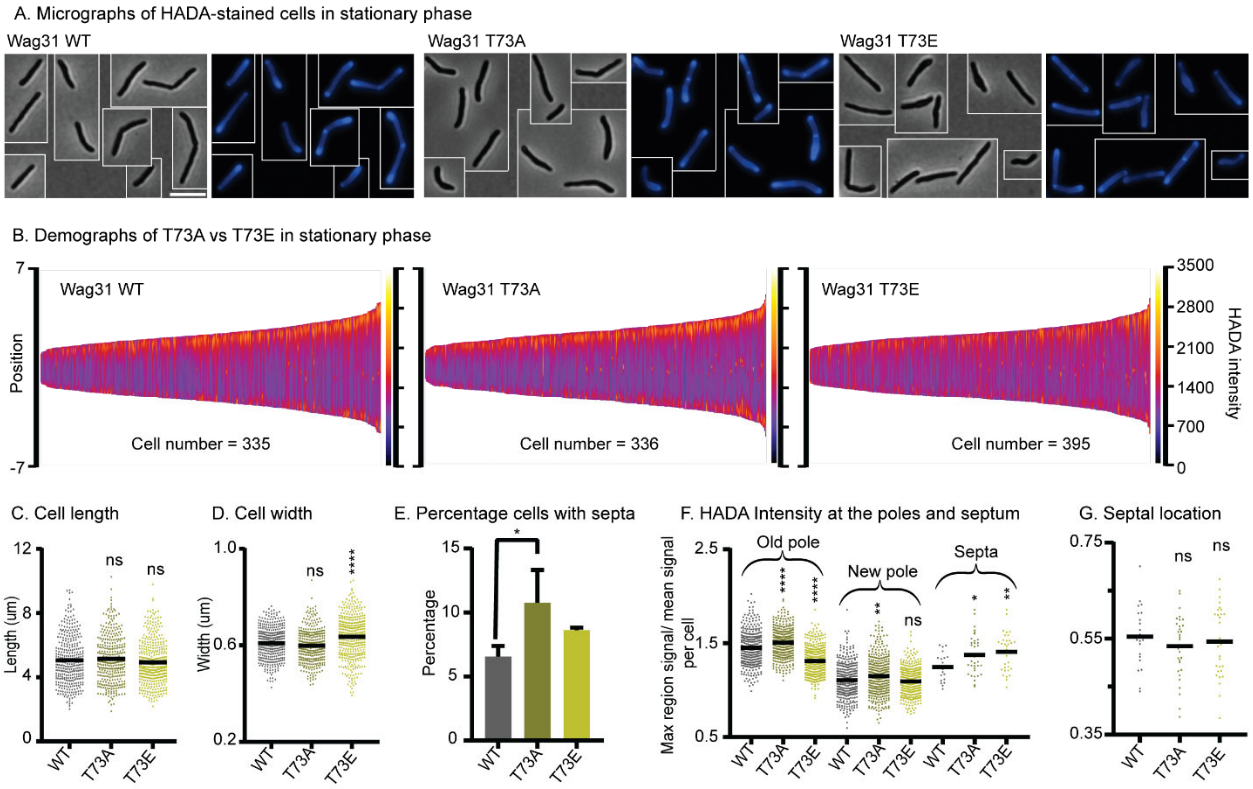

Supplemental Figure 7. Wag31 phospho-site T73 is not an upstream regulator of peptidoglycan metabolism in stationary phase. (A) Phase (left) and fluorescence (right) micrographs of *Msmeg* cells in stationary phase which are expressing Wag31 WT, Wag31 T73A, and Wag31 T73E. (B) Demographs of HADA intensity (color scale) across the length of the cell (Y axis) of the *wag31* allele strains. The cells were sorted by length, with shortest cells on the left and longest on the right of each demograph. Cells were also pole-sorted according to HADA intensity, such that the brighter pole (presumed to be the old pole) was oriented to the top along the Y axis. At least 100 cells were analyzed from each of three independent biological replicates of each strain. (C) Cell lengths of the *wag31* allele strain. Black bars are at the mean. (D) Cell widths of the *wag31* allele strain. Black bars are at the mean. (E) Percentage of cells in (F) that have septal HADA signal. (F) Relative polar and septal HADA intensity of Wag31 WT, Wag31 T73A, and Wag31 T73E. (G) Septal location in *wag31* allele strains. ns, p >0.05, *, P = < 0.05, **, P=< 0.005, ****, P = <0.0001. All *P*-values were calculated by one-way ANOVA, Dunnett's multiple comparisons test.

Demographs of HADA intensity of *wag31 allele strains*

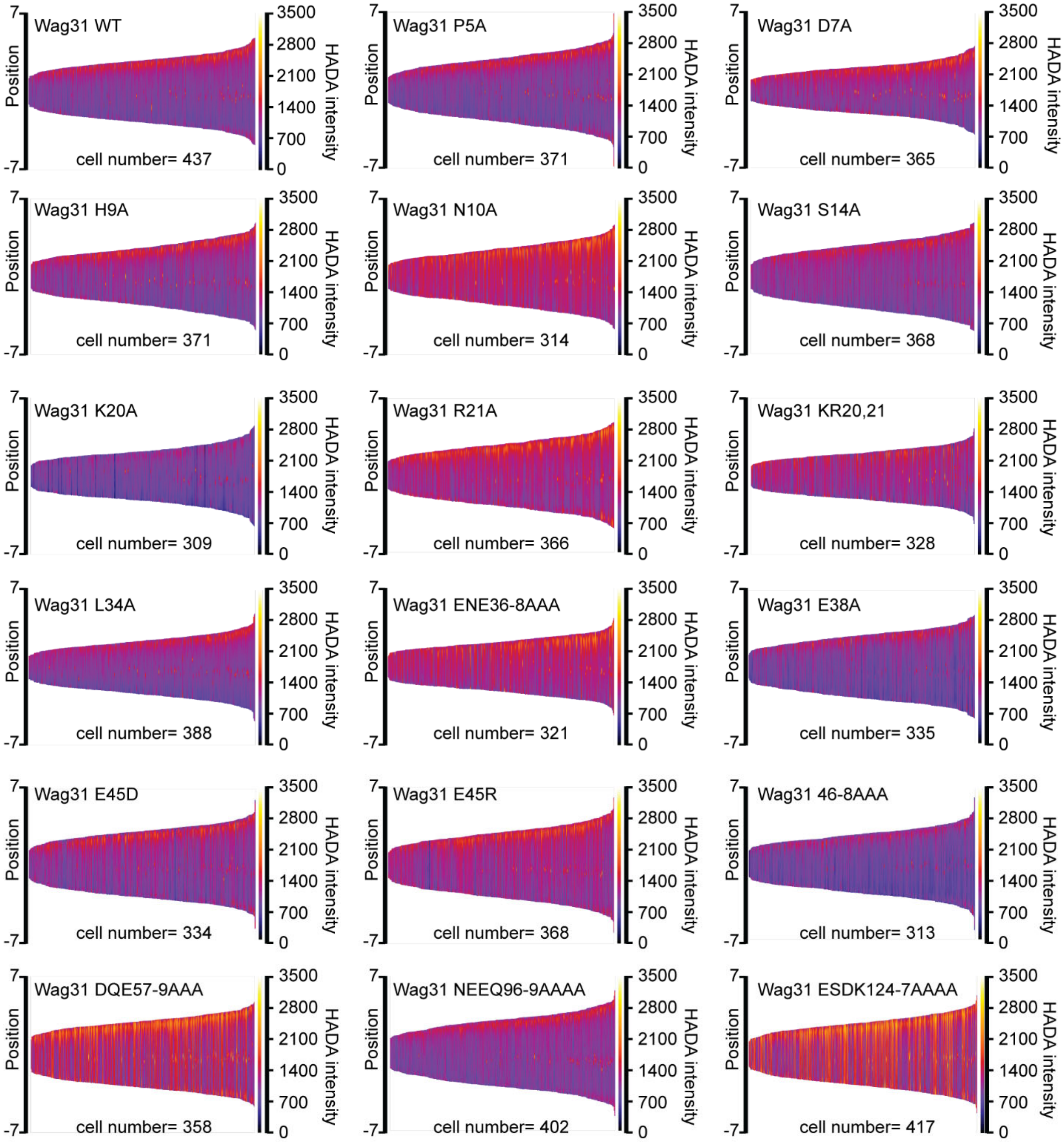

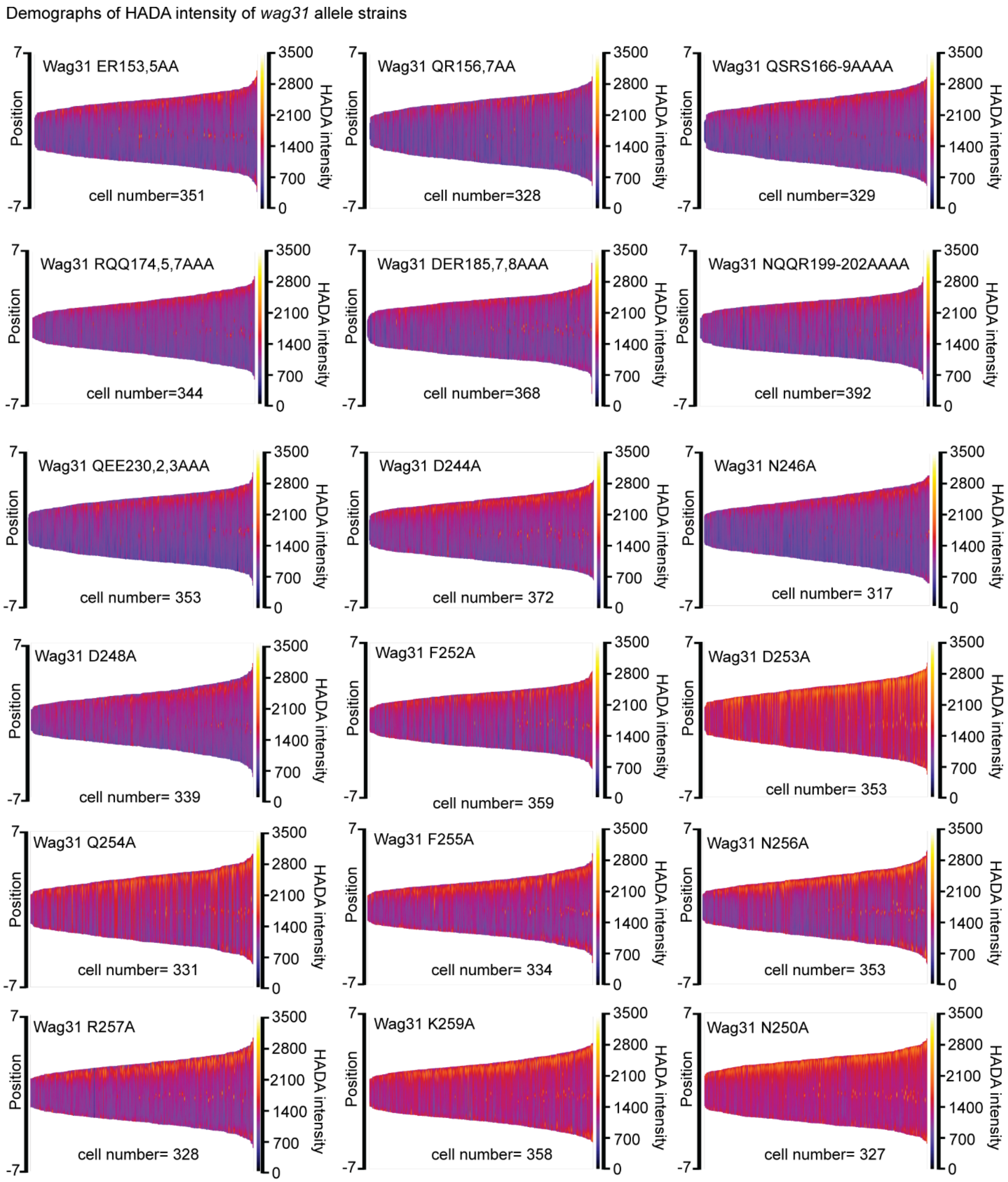

Supplemental Figure 8 and 9. Demographs of HADA intensity (color scale) across the length of the cell (Y axis) of the *wag31* allele strains. The cells were sorted by length, with shortest cells on the left and longest on the right of each demograph. Cells were also pole-sorted according to HADA intensity, such that the brighter pole (presumed to be the old pole) was oriented to the top along the Y axis. At least 100 cells were analyzed from each of three independent biological replicates of each strain.

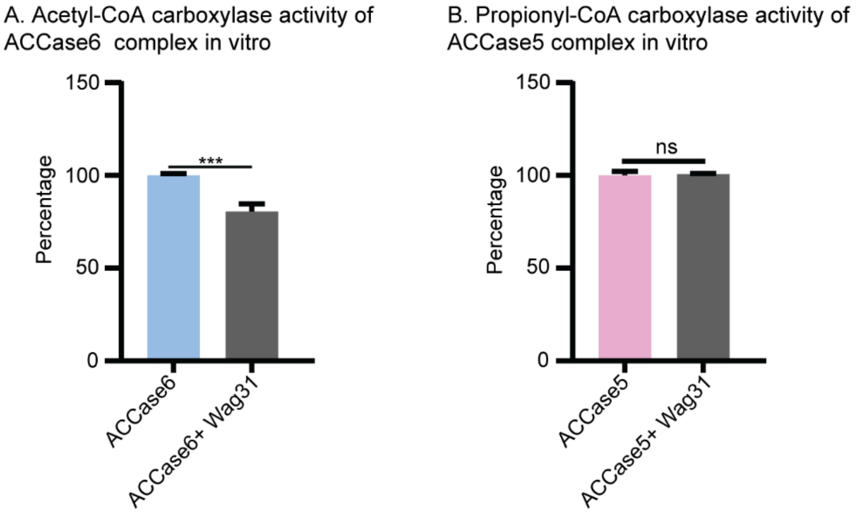

Supplemental Figure 10. *In vitro* activities of the ACCase complexes. ACCase6 and ACCase5 were reconstituted from their purified component and ACC (A) and PCC (B) activity, respectively, was determined in presence of Wag31. Results are the means of three independent experiments ± standard deviations (n = 3). ns, P >0.05, *, P =< 0.05, ***, P=< 0.0005. All *P*-values were calculated by the unpaired t-test.
